# Sodium Alginate‐Based Submucosal Lifting Hydrogel for Endoscopic Mucosal Resection and Endoscopic Submucosal Dissection

**DOI:** 10.1002/adhm.71427

**Published:** 2026-07-11

**Authors:** Iram Maqsood, Venkata S. Akshintala, Surya T. Evani, Rebecca A. Krimins, Yihan Wang, Keith Freel, Ahmed Ibrahim, Saowanee Ngamruengphong, Mouen Khashab, Stephen W. Hoag

**Affiliations:** ^1^ Department of Pharmaceutical Sciences University of Maryland School of Pharmacy Baltimore Maryland USA; ^2^ Division of Gastroenterology Johns Hopkins Hospital Baltimore Maryland USA; ^3^ Russell H. Morgan Department of Radiology and Radiological Science Johns Hopkins University School of Medicine Baltimore Maryland USA

**Keywords:** injectability, lifting agent, mucosal lift persistency, rheological characterization, Sodium alginate, viscosity

## Abstract

Submucosal lifting agents are crucial for enhancing the safety and efficacy of Endoscopic Mucosal Resection (EMR) and Endoscopic Submucosal Dissection (ESD) and other endoscopic interventions involving submucosal tunnelling. Frequently utilized lifting agents include saline, starch, ORISE Gel, Eleview, EverLift, BlueBoost and EndoClot SIS. In this context, we have formulated a sodium alginate‐based lifting gel incorporating biocompatible methylene blue dye, which significantly enhances lesion visualization and delineation. Our Na alginate‐derived lifting hydrogels displayed favorable shear‐thinning characteristics, with an elevated viscosity (44–190 mPa∙s) at lower shear rates compared to commercially available lifting agents (1.7–44 mPa∙s). In lyophilized form, it exhibited short reconstitution time (60–90 s), near physiological pH (∼6.5), and osmolality (265–275 mOsm/kg). Ex vivo assessments validated the sustained lift height and cushion circumference for 60 min, while in vivo investigations in swine illustrated successful injectability, cushion retention for 45 min, and an absence of adverse tissue reactions. The goal was to investigate the relationship between rheological properties and mucosal lifting performance. By integrating prolonged elevation, improved visualization, biocompatibility, and optimal physicochemical characteristics, our alginate‐based lifting gel effectively addresses the limitations inherent in previous agents, offers a more efficient, and versatile next‐generation lifting agent for EMR and ESD.

## Graphical abstract

A biocompatible sodium alginate‐based hydrogel is developed as a next‐generation submucosal lifting agent intended for EMR and ESD. The optimized formulation exhibits a composition devoid of endotoxins, high viscosity, demonstrates reliable injectability, and ensures prolonged mucosal elevation. Both detailed ex vivo and in vivo evaluations substantiate its improved handling, enhanced visualization, and superior procedural performance compared with currently used clinical lifting agents.

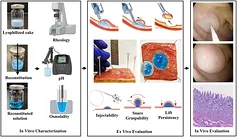

## 1 Introduction

Submucosal lifting agents are indispensable in enabling Endoscopic Mucosal Resection (EMR) [1], and Endoscopic Submucosal Dissection (ESD), which are two minimally invasive methodologies employed for the excision of gastrointestinal mucosal lesions [2, 3]. These agents are administered into the submucosal tissue to elevate the mucosal lesion, thereby creating a delineation from the muscularis propria [4]. This “cushioning” effect serves to safeguard against thermal and mechanical trauma to the underlying tissues while promoting complete lesion excision. Following injection, the lifting agent induces a protrusion that isolates the lesion from the adjacent tissue, thereby enhancing visibility and accessibility for resection [5]. This separation not only reduces the likelihood of perforation but also facilitates the safer application of electrocautery instruments, which are critical for cutting and coagulating throughout the procedures [6]. In general, the commercially available submucosal lifting agents have transformed the safety and effectiveness of EMR and ESD [7].

Currently, there are many commercially available lifting agents on the market, each with advantages and disadvantages that must be judiciously contemplated in clinical use [8]. For example, ORISE Gel, while effective in creating a durable and stable cushion, has been associated with granulomatous foreign body reactions in histological specimens [9, 10, 11]. Nevertheless, Colak et al. documented the formation of a nodule or mass‐like presentation; additionally, the same patient experienced a small bowel obstruction, and subsequently, the excised specimen exhibited serosal fibrosis along with a localized foreign body response with residual ORISE Gel [12, 13]. This can lead to misinterpretation of pathology, mimicking malignancy, and complicating post‐procedural assessment [12, 14, 15]. Additionally, its high viscosity can make injections more challenging, requiring greater force and technical precision [12]. ORISE Gel is also relatively expensive, potentially limiting its accessibility in resource‐constrained settings [9].

Traditional agents like saline are widely used due to their low cost and availability, but they are quickly absorbed, providing only a transient lift and requiring frequent reinjections [6, 8]. Similarly, starch‐based solutions provide a cost‐effective alternative but often offer only short‐term elevation [2, 16]. In general, non‐hypertonic saline is reported to be a safe solution with regard to adverse events and cost‐effectiveness [17]. There have been a number of trials comparing different submucosal injection fluids for ESD use [18, 19, 20, 21]. Ferreira et al. have investigated a number of existing injection solutions in a meta‐analysis, and concluded that sodium hyaluronate and saline injectates were clinically equivalent [22]. Recently, Spadaccini et al. reported that a new biocompatible polymer SIC‐8000 (Eleview) was more effective for ESD and EMR compared to saline solution [23]. Eleview (SIC‐8000) offers better submucosal lift than saline, with the added advantage of dye for improved lesion visualization [24, 25, 26]. However, its elevation effect may not be as long‐lasting as highly viscous agents like sodium hyaluronate, sometimes necessitating reinjections during longer procedures [27]. Cost remains a limiting factor for widespread use [28], and rare allergic reactions to its components have been reported [26]. Mehta et al. found that lower injection volumes and fewer repeat injections were required with starch and Eleview in comparison to saline for the same mucosal lifting effect [16].

EverLift is a pre‐mixed solution containing methylene blue, simplifying preparation and enhancing lesion visibility [29]. However, excessive staining can obscure the dissection plane, complicating the procedure. Its elevation may not last as long as hybrid or highly viscous agents, and availability in some regions is still limited [30]. Significantly, products such as BlueBoost and BlueEye have recently attained approval from the FDA for utilization as submucosal injection materials [29]. BlueBoost is a newer agent that combines elevation with enhanced visualization through incorporated dye. However, excessive staining can interfere with clear visualization of the tissue plane, and its cost can be prohibitive for some healthcare systems [31]. Long‐term safety and efficacy data for BlueBoost remain limited, requiring further studies [29]. LiftUp integrates hybrid properties, combining viscosity and osmotic effects for a durable lift [32]. However, this complexity can demand advanced injection techniques, posing a challenge for less experienced endoscopists [33]. As a newer formulation, it is also relatively costly, and evidence of its long‐term safety and clinical outcomes is still emerging [34]. EndoClot SIS, a starch‐based agent, provides both effective lifting and hemostatic properties, making it especially useful for bleeding‐prone lesions [35, 36]. However, its adhesive nature can make lesion removal more challenging if not injected correctly. Cost and availability can also limit its use in certain regions, and specific training is required for optimal application [37]. Glycerol has been used as submucosal lifting agent for its cost‐effectiveness and high viscosity; however, previous research has highlighted procedural issues with glycerol‐based solutions including histopathological distortion and needs careful administration to avoid tissue damage [38, 39]. Further, glycerol can lead to increased smoke when electrocautery is performed and so it interferes with visualization during the procedure [38, 39]. In summary, while submucosal lifting agents have greatly advanced EMR and ESD procedures, their limitations highlight the need for careful selection based on the clinical context. Balancing cost, effectiveness, and potential side effects is essential, along with adequate operator training to maximize procedural success and minimize complications.

Besides that, underwater EMR/ESD signifies a significant advancement in therapeutic endoscopy. By removing air and introducing saline, the lesion is submerged, enabling enhanced mucosal separation from the muscular layer. The current trend indicates that saline immersion is beneficial as both a rescue and primary method for gastrointestinal resections, particularly in the lower GI tract. Immersion improves endoscope stability, mucosal visualization, and facilitates safe resection by providing physical lifting of mucosal and submucosal layers [40]. However, electrosurgical device activation generates bubbles that hinder visualization [41]. Additionally, bubbles can become trapped in tapered hood attachments, complicating their removal [41]. Employing a higher viscosity agent enhances visualization by preserving the distinction between the lesion and muscularis propria and reducing bubble formation [27]. Moreover, the endoscopic field is evolving toward the resection of larger, more complex lesions. EMR procedures are generally short and involve saline or water, whereas advanced resections, particularly ESD, can last from 45 min to an hour. As endoscopic methods adapt to accommodate larger lesions necessitating prolonged submucosal elevation, we are innovating a sodium alginate‐based lifting gel to aid in these complex procedures.

In this work, various concentrations of sodium alginate were evaluated to formulate an injectable hydrogel capable of facilitating the lifting and dissection of tissue. Herein, we delineate the activities conducted and the outcomes achieved, organizing them into categories of in vitro development and characterization of the formulations in detail, ex vivo assessments of the formulations, and in vivo validation employing a porcine model of gastrointestinal hemorrhage. Then our newly developed sodium alginate‐based lifting gel was compared with commercially available agents in terms of viscosity, pH, osmolality, and ex vivo as well as in vivo endoscopic injectability, lifting persistency, and graspability with snare. Our main objective was to determine the best set of physical and chemical properties of gel for best lifting performance and compare sodium alginate gels with commercially available lifting gels using in vitro, ex vivo, and in vivo methods.

## 2 Results and Discussion

### 2.1 Injectable GI Mucosal Lifting Gels

Injectable gels are formulated for applications in GI tract; they can be used to aid in the dissection or separation of pathological tissue from healthy tissue during surgical resection. The process of “lifting” the lesion establishes a more secure milieu for resection. For the gel to be successfully injected through an endoscope and facilitate the separation of tissues requires a precise combination of physical chemical properties. Thus, we examine the best combination of physical chemical properties of mucosal lifting gel that facilitates the submucosal lifting of a polyp, which eventually assisted in polyp resection. To this end, we have compared the physical and chemical properties of gels made in our lab that have a range of rheological properties with commercially available gels (see Table 1). We have summarized the composition of a typical topical gel formulation in Table S3. In addition, we compared the popular commercially available mucosal lifting gel products on the market in terms of formulation composition, physical and chemical properties, FDA approval date, and shelf life, see Table 2. Also, the physical and chemical properties of our newly developed Na alginate‐based lifting gel are summarized in Table 3. The key point is to identify the physical and chemical properties of the gel that are critical to EMR and ESD performance.

**TABLE 1:** Newly developed sodium alginate‐based lifting gel formulations including the source of sodium alginate and the in vitro, in vivo and ex vivo tests performed and the commercially available lifting agents studied.

	Source	Acronym	Conc. [% w/v]	In vitro Characterization	Ex vivo Evaluation	In vivo Study
Sodium alginate	VIVAPHARM Alginate PHU 175 (JRS Pharma, France)	JRS Na Alg	0.6%	Lyophilization, Cake appearance, Reconstitution time, Color (visual), Rheology, pH, Osmolality, Injectability	Submucosal lifting, Efficacy testing in an intact stomach	
			0.8%			Mucosal lifting, Safety study
			1%			
	Pronova UP MVG (NovaMatrix)	NovaMatrix MVG	0.8%	Lyophilization, Cake appearance, Reconstitution time, Color (visual), Rheology, pH, Osmolality, Injectability		
			1%		Submucosal lifting, Efficacy testing in an intact stomach	Mucosal lifting, Safety study
	Pronova UP MVM (NovaMatrix)	NovaMatrix MVM	0.8%	Lyophilization, Cake appearance, Reconstitution time, Color (visual), Rheology, pH, Osmolality, Injectability		
			1%		Submucosal lifting, Efficacy testing in an intact stomach	
**Commercially Available Lifting Agents**						
Saline	BD PosiFlush			Color (visual), Rheology, pH, Osmolality, Injectability	Submucosal lifting, Efficacy testing in an intact stomach	Mucosal lifting
Starch (HEXTEND)	Hospira Inc.				−	
ORISE Gel	Boston Scientific				−	
Eleview	Cosmo Pharmaceutical NV				−	
EverLift	Laborie Medical Technologies				−	
BlueBoost	Microtech Endoscopy				−	
Liftal K	Kaigen Pharma				−	

**TABLE 2:** Properties of commercially available lifting formulations, including the most popular products on the market.

Compound and company	FDA approval date	Viscosity Modifier(s)	Buffer	Preservative	Osmotic agent	Vehicle	Contrast agent	pH	Osmolality [mOsm/kg]	Storage [°C]	Shelf life
Saline (BD PosiFlush, NJ, USA)					NaCl	Water	Colorless	7.29	286.3 ± 0.5	^c)^RT	2 years
Starch (HEXTEND, Hospira Inc. Lake forest IL)		6% HetaStarch			NaCl Sodium lactate KCl Dextrose CaCl_2_ MgCl_2_	Water for Injection	Colorless	6.21	307 ± 0.8	^d)^RT	2 years
ORISE Gel (Boston Scientific)	September 2018	Gellan gum Polysaccharide			NaCl	Water for Injection	FD&C blue No. 1	6.15	289.7 ± 0.47	4 – 25	^a)^NA
Eleview (Cosmo Pharmaceuticals)	September 2015	Poloxamer 188 Polyoxyl‐15‐hydroxystearate Medium chain triglycerides	—	—	NaCl	Water for Injection	Methylene blue	6.37	279.7 ±1.25	2 – 25	2 years
EverLift (Laborie Medical Technologies)	July 2020	Hydroxyethyl Cellulose Glycerin	Na phosphate K phosphate	Benzyl alcohol		Water	Methylene blue	7.67	3)^c)^	(15 – 30	2 years
BlueBoost (Micro‐Tech Endoscopy)	June 2021	Na hyaluronate	^b)^Na_2_HPO_4_, NaH_2_PO_4_		NaCl	Water for Injection	Methylene blue	6.87	288.3 ± 0.94	1 – 30	2 years
LiftUp (Ovesco)	August 2022	Poloxamers			NaCl	Water	Methylene blue			2 – 8	^a)^NA
EndoClot SIS (Olympus)	April 2020	Absorbable Starch polymers			Mix Powder with Saline ^c)^(draw using spiral syringe)		Colorless	7.00		^d)^RT	2 years
Liftal K (Kaigen Pharma)	November 2018 (^c)^Japanese)	Sodium alginate	Na_2_HPO_4_, NaH_2_PO_4_		NaCl	Water	Colorless	7.40		1 – 30	3 years

^a)^ NA = Not Available.

^b)^ NaH_2_PO_4_ is monosodium phosphate, also known as sodium dihydrogen phosphate, and Na_2_HPO_4_ is dibasic sodium phosphate, also known as disodium hydrogen phosphate.

^c)^ Unable to measure osmolality of EverLift, solution did not freeze in temperature range of osmometer probably because of the presence of other additives such as glycerin.

^d)^ RT = Room Temperature.

**TABLE 3:** Composition and properties of our newly developed sodium alginate‐based lifting gels.

Compound and company	Viscosity Modifier	G/M ratio	Conc. [% w/v]	Buffer	Contrast agent	pH	Osmolality [mOsm/kg]	Storage (lyophilized cake) [°C]
JRS Na Alg	Sodium alginate	≤ 0.67	0.6%	^a)^PBS (0.8X, pH 6.5)	Methylene blue (0.5 mg / 100 mL)	6.50	269.7 ± 1.2	1 – 30
			0.8%			6.50	283.0 ± 0.8	
			1%			6.50	286.3 ± 0.5	
NovaMatrix MVG		≥ 1.5	0.8%			6.50	267.0 ± 0.8	
			1%			6.50	271.7 ± 1.7	
MVM NovaMatrix		≤ 1	0.8%			6.50	282.3 ± 2.1	
			1%			6.50	290.3 ± 0.5	

^a)^ Phosphate buffered saline comprises Na_2_HPO_4_ (anhydrous), KH_2_PO_4_ and NaCl.

### 2.2 Rheological Characterization of the Lifting Gels

Viscosity is a key property of a hydrogel that determines its effectiveness as a sub‐mucosal lifting agent when injected under a polyp. The ∼0.24 mm narrow internal diameter of a, typically used, 25‐gauge endoscopy injection needle creates an upper limit of gel viscosity as injection pressures become too large to be used practically during a procedure. However, if the viscosity is too low the extent and rate at which the gel disperses between the tissue layers becomes too fast and multiple injections are required during a procedure which increases procedure time. In addition, if the gel is too viscous it can make it more difficult to snare during polyp resection.

Alginate gel viscosity is dictated by the molecular weight and the M to G ratio. Thus, when characterizing a gel, one must account for molecular weight and the M to G ratio. For our studies we used 3 grades of alginate. Specifically, two grades of sodium alginate were sourced from NovaMatrix and one from JRS Pharma. NovaMatrix (an IFF brand) manufactures high‐purity, biocompatible sodium alginates, notably high‐G (MVG) and high‐M (MVM) variants, for biomedical applications such as cell encapsulation, 3D bioprinting, and drug delivery. According to Na alginate manufacturer's product literature JRS Na Alg has M:G ratio of between 1.0–1.5:1.0, NovaMatrix MVG has a G/M ratio ≥ 1.5 and NovaMatrix MVM has a G/M ratio ≤ 1 (see Table 3). Both Pronova and VIVAPHARM alginates have a MW of around 200 kDa.

**FIGURE 1:**
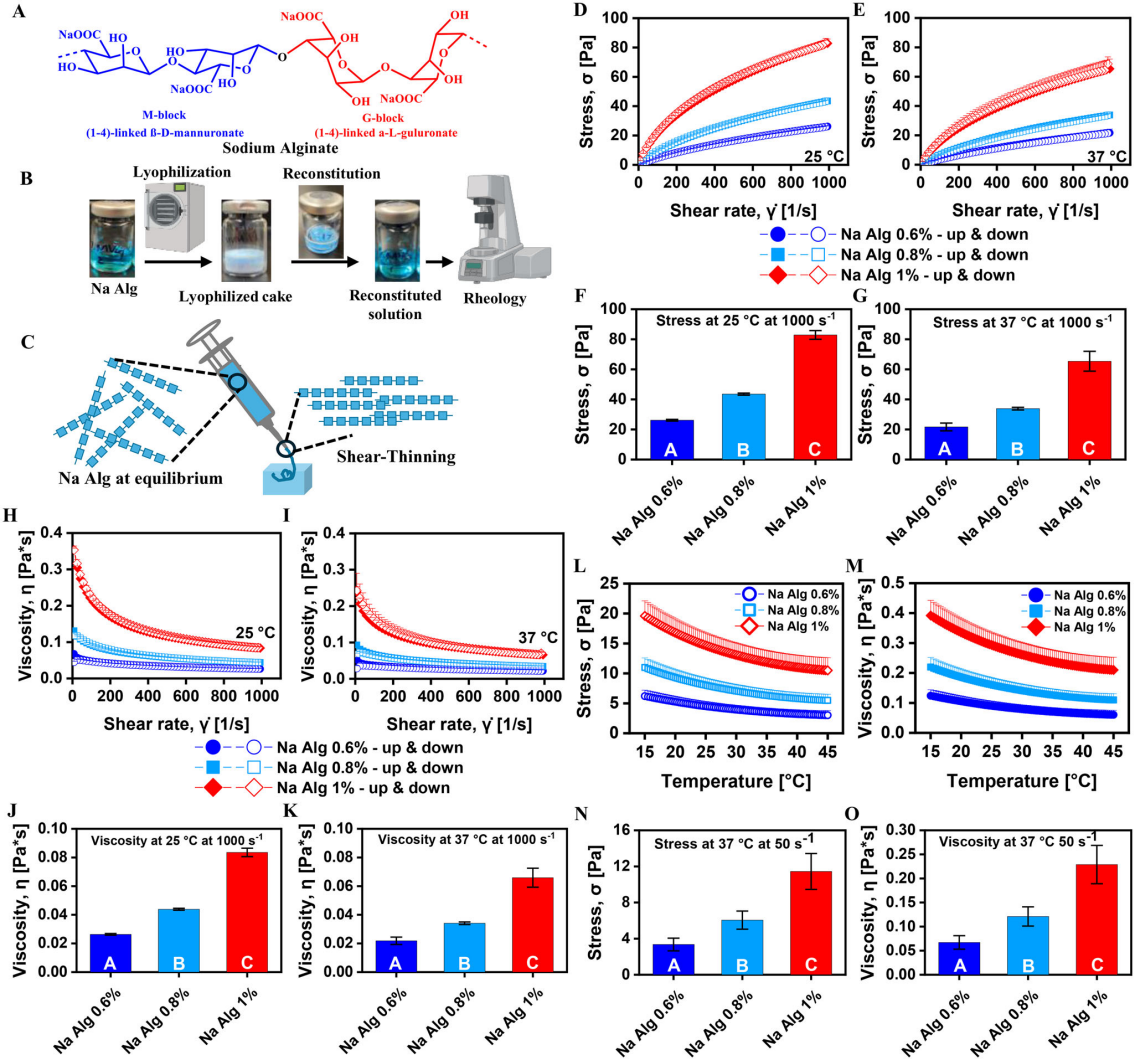
Preparation and rheological characterization of JRS Na Alg‐based lifting gel. [A] Chemical structure of Na Alginate, [B] Preparation of JRS Na Alg‐based lifting gel, and [C] Illustration of shear thinning behavior. [D] and [E] Graphs represent the rheological properties of the JRS Na Alg solution of different concentrations, namely, 0.6%, 0.8%, and 1% in PBS of pH 6.5 with MB measured at 25°C and 37°C respectively as a function of shear rate and shear stress. Empty symbols, up curve; closed symbols, down curve (n = 3). For reasons of clarity, the last points of the stress profiles at highest shear rate of 1000 s^−1^ are plotted separately at [F] 25°C and [G] 37°C. [H] and [I] Graphs represent viscosity of the JRS Na Alg solution of different concentrations, namely, 0.6%, 0.8%, and 1% as a function of shear rate at 25°C and 37°C respectively. The last points of the viscosity profiles are plotted separately at [J] 25°C and [K] 37°C. [L]and [M] Stress and viscosity of JRS Na Alg 0.6%, 0.8%, and 1%, as measured using temperature ramp at 15°C to 45°C at constant shear rate of 50 s^−1^. [N] and [O] Stress and viscosity values of each formulation at 37°C are presented. Data points and columns with error bars represent mean ± standard deviations (*n* = 3). Columns with different letters are statistically significantly different (*p* < 0.05).

The rheological characteristics were evaluated at 25°C and 37°C (Figure 1). The stress and viscosity of JRS Na Alg was measured at 0.6%, 0.8%, and 1% (w/v) concentrations, and shear rates ranging from 0 to 1000 s^−1^ (see Figure 1D–G (shear stress) and H–K (viscosity)). Also, the results at shear rates of 142 s^−1^ and 1000 s^−1^ are summarized in Table 4 while graphs Figure 1L–O show the viscosity and shear stress as a function of temperature (15°C to 45°C). Viscosity decreased with increase of temperature. As expected, these gel systems exhibited shear‐thinning, a characteristic of non‐Newtonian rheological behavior. These results showed that the viscosity of JRS Na Alg increases in a concentration‐dependent manner, with the 0.6% solution exhibited the lowest viscosity and the 1% solution demonstrated the highest viscosity (see Figure 1). Also, the viscosity of JRS Na Alg was assessed with and without methylene blue, revealing no change in viscosity or injectability at the tested concentration. In addition to this, while we were formulating gel for injectable applications, our objective was to evaluate the sodium alginate that is devoid of endotoxins, and the sodium alginate sourced from JRS did not satisfy these specifications.

**TABLE 4:** Viscosity profile of different concentrations of JRS Na Alg 0.6%, 0.8% and 1% and NovaMatrix MVG and NovaMatrix MVM at 25°C and 37°C at two different shear rates.

Sodium alginate (source)	Concentration [% w/v]	Temperature [°C]	^a)^Viscosity ± S.D. [mPa∙s]	^b)^Viscosity ± S.D. [mPa∙s]
JRS Na Alg	0.6%	25°C	26.36 ± 0.58	44.65 ± 0.94
	0.8%		43.87 ± 0.69	84.55 ± 0.78
	1%		83.54 ± 2.93	192.4 ± 3.39
	0.6%	37°C	21.85 ± 2.57	35.16 ± 4.53
	0.8%		34.11 ± 0.87	62.49 ± 1.88
	1%		65.9 ± 6.63	138.6 ± 10.7
NovaMatrix MVG	0.8%	25°C	52.54 ± 1.45	88.12 ± 2.03
	1%		61.18 ± 2.33	110.99 ± 1.26
NovaMatrix MVM	0.8%		49.64 ± 0.34	87.81 ± 1.36
	1%		62.13 ± 1.62	121.38 ± 3.17
NovaMatrix MVG	0.8%	37°C	46.58 ± 2.02	70.47 ± 3.21
	1%		47.77 ± 1.91	75.58 ± 3.60
NovaMatrix MVM	0.8%		41.87 ± 0.12	66.98 ± 0.35
	1%		50.18 ± 0.29	86.89 ± 0.54

^a)^ Viscosity as measured at 1000 s^−1^ shear rate.

^b)^ Viscosity as measured at 142 s^−1^ shear rate.

**FIGURE 2:**
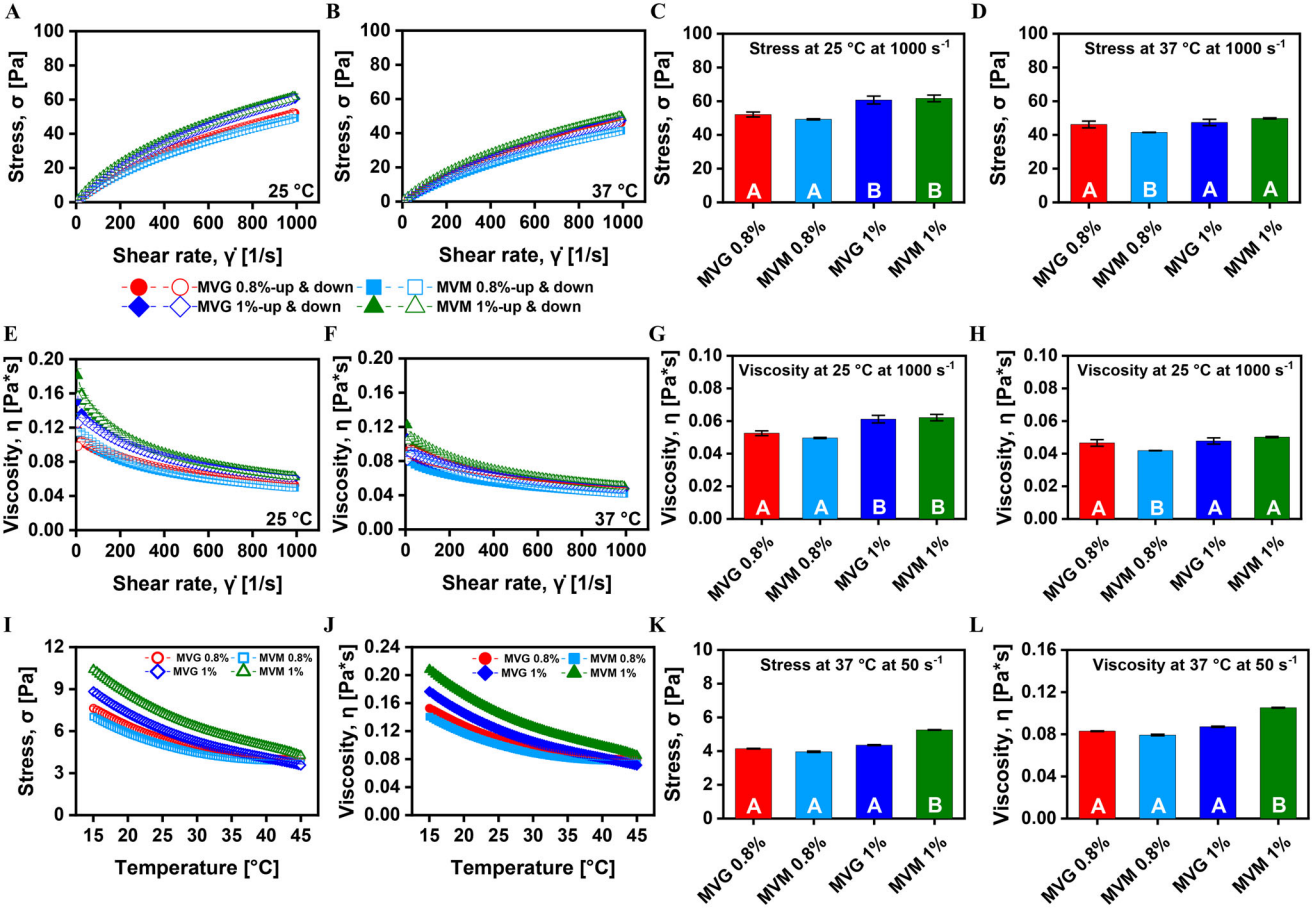
Preparation and rheological characterization of NovaMatrix MVG and NovaMatrix MVM ‐based lifting gel. [A]and [B] Graphs represent the rheological properties of NovaMatrix MVG and NovaMatrix MVM 0.8% and 1% (in 0.8 X PBS of pH 6.5 with methylene blue) as a function of shear rate and shear stress at 25°C and 37°C. Empty symbols, up curve; closed symbols, down curve. For reasons of clarity, the last points of the stress profiles at the highest shear rate of 1000 s^−1^ are plotted separately at [C] 25°C and [D] 37°C. [E] and [F] Graphs represent viscosity as a function of shear rate at 25°C and 37°C, respectively. The last points of the viscosity profiles are plotted separately at [G] 25°C and [H] 37°C. [I]and [J] Stress and viscosity of NovaMatrix MVG and NovaMatrix MVM 0.8% and 1%, respectively, measured using temperature ramp at 15°C to 45°C at constant shear rate of 50 s^−1^. [K] and [L] Stress and viscosity values of each formulation at 37°C are presented. Data points and columns with error bars represent mean ± standard deviations (*n* = 3). Columns with different letters are statistically significantly different (*p* < 0.05).

Next, we compared ultra‐pure endotoxin free NovaMatrix MVG and NovaMatrix MVM at concentrations of 0.8% and 1% w/v. The viscosities were evaluated at 25°C and 37°C and shear rates ranging from 0 to 1000 s^−1^. The results are shown in Figure 2 and the results at shear rates of 142 and 1000 s^−1^ are summarized in Table 4. The stress profiles of the NovaMatrix MVG‐ and NovaMatrix MVM‐based lifting gels are shown in Figure 2, panels A–D; viscosity profiles are presented in panels E–H at 25°C and 37°C respectively; and shear stress and viscosity as functions of temperature are shown in panels I–L (see Figure 2). The viscosity of Na alginate increased in a concentration‐dependent manner and the viscosities extracted from at shear rates of 142 s^−1^ and 1000 s^−1^ are shown in Table 4. As with the JRS Na‐Alg, the data shows non‐Newtonian shear thinning behavior. In addition, we didn't find any significant differences in viscosity between NovaMatrix MVG or NovaMatrix MVM at the concentrations of 0.8% and 1% and at temperatures of 25°C and 37°C, as determined by three‐way ANOVA (*p* > 0.05). Likewise, no significant differences were observed in the three‐way interactions between JRS Na Alg at 0.8% and NovaMatrix MVG or NovaMatrix MVM at the concentrations of 0.8% and 1% and at temperatures of 25°C and 37°C at shear rates of 142 s^−1^ and 1000 s^−1^. Conversely, all remaining concentrations (JRS Na Alg 0.6% and 1%) exhibited statistically significant differences (*p* < 0.05) as well as compared with NovaMatrix MVG and NovaMatrix MVM. Thus, when flowing through endoscopic tubing the viscosity decreases and when at rest the viscosity increases, which facilitates submucosal lifting. Overall, rheological analyses indicated that JRS Na Alg 0.6% matched the viscosity of 0.8% NovaMatrix Na Alginate, whereas JRS Na Alg 0.8% was equivalent to 1% NovaMatrix; notably, JRS Na Alg 0.8% and 1% NovaMatrix exhibited optimal and sustained lift persistence.

Sodium alginate solutions are not stable at room temperature for longer periods of time due to microbial growth and polymer degradation; thus, we studied the lyophilization of Na alginate solutions to see how the lyophilization process affects the viscosity of reconstituted solutions. Thus, we determined the viscosity of NovaMatrix MVG 1% and NovaMatrix MVM 1% before and after lyophilization. These studies didn't find any significant differences in viscosity profiles before or after lyophilization (data not shown); we found the viscosity curves nicely overlapped. This is important as lyophilization is one of the best ways to extend the product shelf‐life.

**FIGURE 3:**
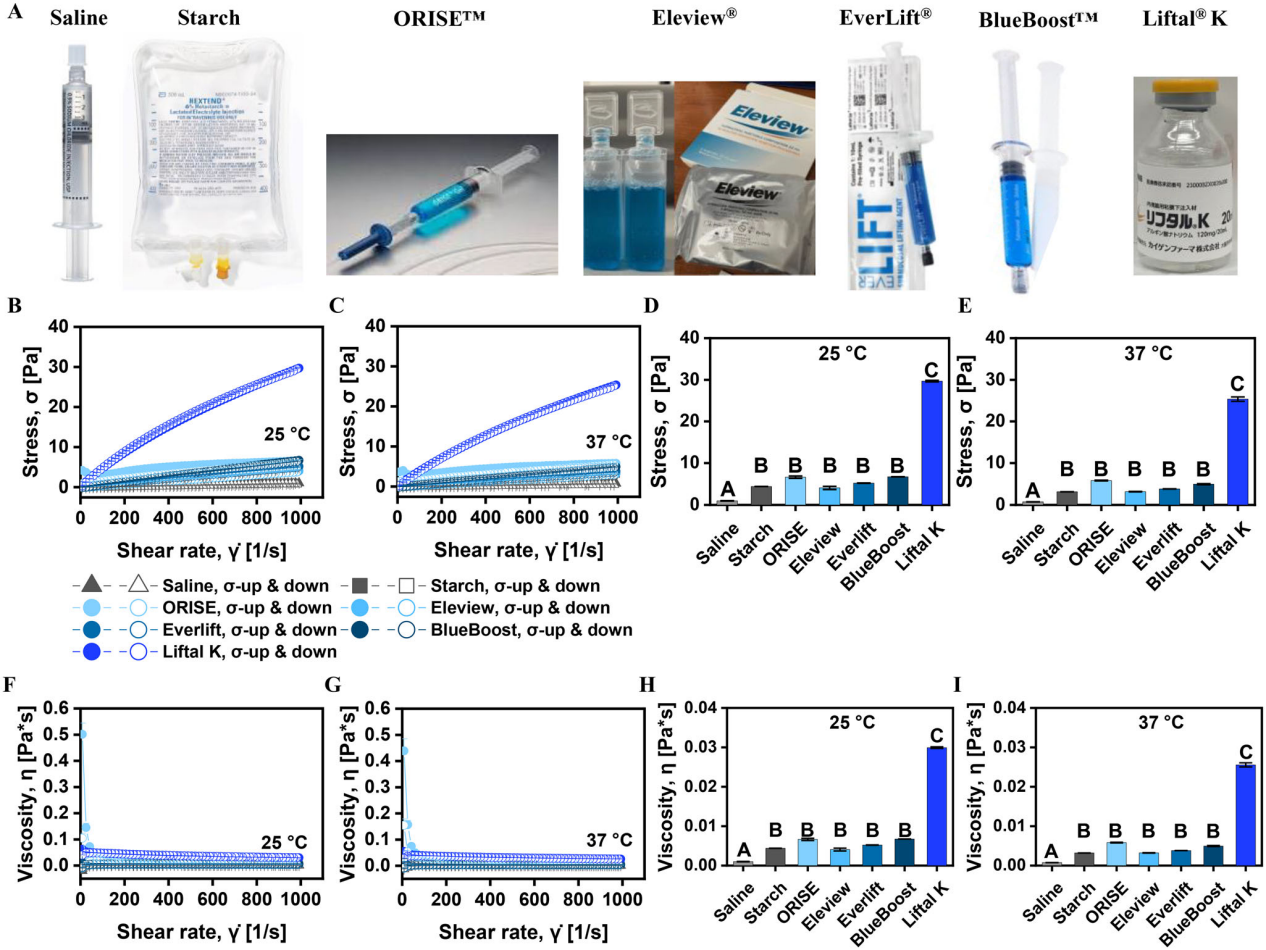
Rheological characterization of commercially available mucosal lifting agents. [A] Controls (saline, starch) and Liftal K are transparent or without dye while ORISE Gel, Eleview, EverLift, BlueBoost are blue in color. [B], [C] Graphs represent the rheological properties of marketed lifting gels, for example, ORISE Gel, Eleview, EverLift, BlueBoost, Liftal K in comparison to controls 7 Saline and Starch, as a function of shear rate and shear stress at 25°C and 37°C. Empty symbols, up curve; closed symbols, down curve. For reasons of clarity, the last points of the stress profiles at highest shear rate of 1000 s^−1^ are plotted separately at [D] 25°C and [E] 37°C. [F] and [G] Graphs represent viscosity as a function of shear rate at 25°C and 37°C, respectively. The last points of the viscosity profiles are plotted separately at [H] 25°C and [I] 37°C. Data points and columns with error bars represent mean ± standard deviations (*n* = 3). Columns with different letters are statistically significantly different (*p* < 0.05).

The rheological properties of commercially available lifting gels for example, ORISE Gel, Eleview, EverLift, BlueBoost and Liftal K in comparison to controls, specifically, saline and starch, was measured at 25°C and 37°C and the results obtained were summarized in Figure 3 and Table 5. As shown in Figure 3B–E Eleview, EverLift and BlueBoost showed pseudoplastic behavior. Neither of these products displayed significant hysteresis. The ORISE Gel was quite different. As seen in the upcurve, there was some initial structure that broke down as the shear rate increased from 0 to 1000 s^−1^. This structure was not recovered while shear continues, as evidenced by the linear down curve. Viscosity of commercially available lifting agents at 25°C and 37°C was determined as shown in F–I. Viscosity was calculated in mPa∙s at 142 and 1000 s^−1^ shear rate in order to determine the shear thinning behavior as shown in Table 5. Liftal K represented the highest viscosity of all marketed lifting gel products with 44.94 mPa∙s ± 0.43 and 37.02 mPa∙s ± 0.95 at 142 s^−1^ shear rate while 29.93 mPa∙s ± 0.2, and 25.55 mPa∙s ± 0.52 at 1000 s^−1^ shear rate at 25°C and 37°C, respectively. All the gels represented shear‐thinning behavior except saline and Eleview while none of these products were highly viscous.

**TABLE 5:** Viscosity (1 mPa∙s = 1 cP) comparison of marketed lifting gels: Saline, starch, ORISE Gel, Eleview, EverLift, BlueBoost and Liftal K at 25°C and 37°C at two different shear rates.

	Temperature [°C]	^a)^Viscosity ± S.D. [mPa∙s]	^b)^Viscosity ± S.D. [mPa∙s]
Saline	25°C	1.003 ± 0.004	1.628 ± 0.002
Starch		4.39 ± 0.01	5.05 ± 0.04
ORISE Gel		6.64 ± 0.29	23.42 ± 0.88
Eleview		4.07 ± 0.36	4.72 ± 0.35
EverLift		5.21 ± 0.05	7 ± 0.07
BlueBoost		6.74 ± 0.03	7.78 ± 0.04
Liftal K		29.93 ± 0.2	44.94 ± 0.43
Saline	37°C	0.858 ± 0.005	1.47 ± 0.001
Starch		3.19 ± 0.01	3.83 ± 0.02
ORISE Gel		5.8 ± 0.1	21.9 ± 0.6
Eleview		3.22 ± 0.05	3.88 ± 0.05
EverLift		3.81 ± 0.02	5.11 ± 0.04
BlueBoost		4.95 ± 0.13	6.59 ± 0.85
Liftal K		25.55 ± 0.52	37.02 ± 0.95

^a)^ Viscosity as measured at 1000 s^−1^ shear rate.

^b)^ Viscosity as measured at 142 s^−1^ shear rate.

### 2.3 Mathematical (Power Law) Model Fitting on the Rheological Data of the Lifting Gels

All samples (except saline) demonstrated shear‐thinning; to characteristics the rheological behavior of the samples, the curves were fitted using log transformation of Power‐law Equation (see Equation 2). The Power‐law model provided an adequate fit, with the regression coefficient (R^2^) varying from 0.990 (Eleview) to 0.999 (JRS Na Alg 0.6% and Liftal K) for all tested lifting gels formulations except 0.982 (saline) and 0.861 (ORISE Gel), see Table 6. Although Saline exhibits Newtonian behavior, the incorporation of methylene blue may have modified its rheological behavior, leading to a value of n not equal to 1, which is an indication of non‐Newtonian behavior [42, 43]. Importantly, Na alginate‐based lifting gels’ R^2^ values varied from 0.995 to 0.999 representing that Power Law model is suitable mathematical approach to describe their viscosity pattern. The consistency index (K) and flow behavior index (n) from the log transformation of Equation (2) were determined for each solution using MATLAB's built in curve fitting algorithm.

**TABLE 6:** Quantifying shear thinning behavior using the Power Law model (Equation 1) for lifting gels that were tested via a ramp up curve at 25°C with shear rates going from 1 to 1000 s^−1^. The table gives Power Law parameter estimates for the fitted parameters.

	^a)^ *n*	*Log K*	^b)^ *K*	^c)^ *R^2^*
			(Pa∙s^n^)	
Saline	0.928 ± 0.005	−2.779 ± 0.016	0.002 ± 0.000	0.982
Starch	0.819 ± 0.05	−1.854 ± 0.015	0.014 ± 0.000	0.992
ORISE Gel	0.216 ± 0.015	0.141 ± 0.051	1.390 ± 0.158	0.861
Eleview	0.804 ± 0.030	−1.849 ± 0.036	0.014 ± 0.001	0.990
EverLift	0.796 ± 0.001	−1.687 ± 0.005	0.021 ± 0.000	0.997
BlueBoost	0.857 ± 0.002	−1.765 ± 0.008	0.017 ± 0.000	0.996
Liftal K	0.836 ± 0.002	−1.009 ± 0.002	0.098 ± 0.001	0.999
JRS Na Alg 0.6%	0.780 ± 0.001	−1.896 ± 0.012	0.127 ± 0.004	0.999
JRS Na Alg 0.8%	0.729 ± 0.002	−0.516 ± 0.002	0.305 ± 0.002	0.997
JRS Na Alg 1%	0.643 ± 0.008	0.021 ± 0.010	1.050 ± 0.025	0.995
NovaMatrix MVG 0.8%	0.802 ± 0.004	−0.653 ± 0.008	0.222 ± 0.004	0.998
NovaMatrix MVG 1%	0.771 ± 0.012	−0.492 ± 0.017	0.322 ± 0.013	0.996
NovaMatrix MVM 0.8%	0.772 ± 0.010	−0.589 ± 0.031	0.258 ± 0.018	0.998
NovaMatrix MVM 1%	0.733 ± 0.001	−0.372 ± 0.016	0.425 ± 0.016	0.996

^a)^ *n* is slope, or *n* is the power law index of the shear rate.

^b)^ *K* is known as the consistency coefficient and can be associated directly with viscosity.

^c)^ The correlation coefficient (R^2^).

As JRS Na Alg concentration increased from 0.6% to 1%, K values rose, reflecting higher viscosity. Concurrently, n values showed a slight decline, indicating increased shear‐thinning behavior under applied stress (see Table 6). Overall, parameter K is designated as the consistency coefficient and can be correlated with the viscosity characteristics of the polymeric system. It is noteworthy that the highest K value for ORISE Gel (1.390) indicated initially high viscosity and then immediately viscosity dropped with shear or highest shear thinning while having the second highest K value was observed for JRS Na Alg 1% (1.050) in our investigation, implying a substantial intermolecular linkage within the alginate matrix. The parameter *n* denotes the power law index associated with the shear rate [44]. It was evident that all tested lifting gel formulations represented shear thinning behavior with value of *n* between 0 to 1 (see Table 6). A significant characteristic noted in these samples is the value of *n*, which varies from 0.216 lowest value (ORISE Gel) indicating the most shear thinning (that was also evident in C and D) to 0.928 highest value (saline), suggesting low to no shear thinning; also, indicating Saline's Newtonian behavior. It is important to mention here that JRS Na Alg 1% showed the second highest shear thinning behavior out of all tested lifting gel formulations with *n* value of 0.643 that was also obvious in Figure 1H,I.

### 2.4 pH and Osmolality of Lifting Gel

Injectable hydrogels are a class of biomaterials widely used in various biomedical applications, including drug delivery, tissue engineering, and regenerative medicine [45]. The pH and osmolality of injectable hydrogels are crucial parameters that can significantly affect their biocompatibility, stability, and performance in vivo [46]. In addition, the pH plays a critical role in modulating cellular responses, drug release kinetics, and overall biocompatibility [47]. To minimize tissue irritation and inflammation upon injection, the pH of injectable hydrogels ideally should be maintained within physiological ranges, which are typically between pH 6.5 to 7.4 [48]. However, some drugs are not stable at physiological pHs; in such cases, the formulator must balance between ensuring biocompatibility and maintaining drug stability.

To examine the range of pHs in commercially available injection gels we measured the pH of seven popular products, see Table 2. The pH of commercially available lifting gels e.g., saline, starch, ORISE Gel, Eleview, EverLift, and BlueBoost were between 6.15 (ORISE Gel) to 7.67 (EverLift). In addition, we measured the pH of our in‐house gels created for this study, see Table 3 and we found an average pH value of 6.5 of our gels. Schneider et al. reported pH values for the infusion solutions ranged from 3 to 7.5 and further stated that all solutions had a pH lower than 6, except dexamethasone‐containing solutions (pH 7.3) [49]. This study corroborated the investigation of Oliver et al. where they reported the local reactions at infusion sites, confirmed by histopathology, and concluded that these reactions might be due to lower‐than‐neutral pHs [50]. According to the literature, a neutral pH, with a range between 6.5 and 7.5 is preferred for injectable and subcutaneous infusion solutions in order to reduce skin reactions [49, 50].

Osmolality refers to the concentration of osmotically active solutes in a solution and influences the osmotic pressure across cell membranes. Injectable hydrogels with inappropriate osmolality levels can cause cellular damage and dysregulation. Therefore, it is essential to carefully control the osmolality of injectable hydrogels to ensure compatibility with surrounding tissues and cells. Osmolality of commercially available lifting gels e.g., Saline, Starch, ORISE Gel, Eleview, EverLift, and BlueBoost are given in Table 2. The osmolality of JRS Na Alg 0.6%, 0.8% and 1%, NovaMatrix MVG and NovaMatrix MVM 0.8% and 1% respectively in phosphate‐buffered saline (PBS 0.8X) containing methylene blue (MB) measured using automatic Osmometer (OSMETTE S) was given in Table 3. Dang et al. reported an isotonic (300 mOsm·L^−1^) injectable hydrogel resulted in better cell viability of encapsulated cells than hypertonic injectable hydrogel indicating that isotonicity is an important factor for cellular encapsulation and tissue engineering applications of injectable hydrogels [51]. Similarly, in another study by Schneider et al. (1997), reported the osmolality of subcutaneous drug infusion solutions ranged from of 195 to 294 mOsm/kg [49], while Aragona et al. (2019) found osmolality of hyaluronic acid–based lubricant eye drops to be within the range of 154 to 335 mOsm/kg [52, 53]. According to Rasouli, the physiological osmolality of human plasma is approximately 280–300 milliosmoles per kilograms (mOsm/kg) [54]. This range may vary slightly depending on factors such as hydration status, electrolyte balance, and individual variation, but it generally remains within this narrow window to support normal physiological function. Overall, the osmolality of injectable Na alginate‐based lifting hydrogels was controlled and adjusted to < 300 mOsm/kg (Table 3). In conclusion, the pH and osmolality of sodium alginate injectable hydrogels was carefully controlled to ensure their biocompatibility and efficacy in biomedical applications, which were found 6.5 (pH) and between 267 – 289 mOsm/kg.

### 2.5 Color of Lifting Gel

Injectable lifting hydrogels can be formulated with dyes to impart a distinct coloration; for example, FD&C Blue No. 1 (Brilliant Blue FCF), FD&C Blue No. 2 (Indigo Carmine) and Methylene Blue (phenothiazine derivative) are commonly used blue dyes that are considered safe for use in medical and food applications, see Table 2. The addition of methylene blue dye to injectable hydrogels improves their visibility during surgical procedures. The blue coloration provides surgeons with clear visual cues, aiding in precise placement and localization of the hydrogel at the target site. This enhanced visibility can be particularly beneficial in applications such as tissue engineering, wound healing, and drug delivery [55]. The color of the gel is a function of the amount of dye added and color consistency is a critical quality attribute; thus, color evaluation is important for product consistency.

**FIGURE 4:**
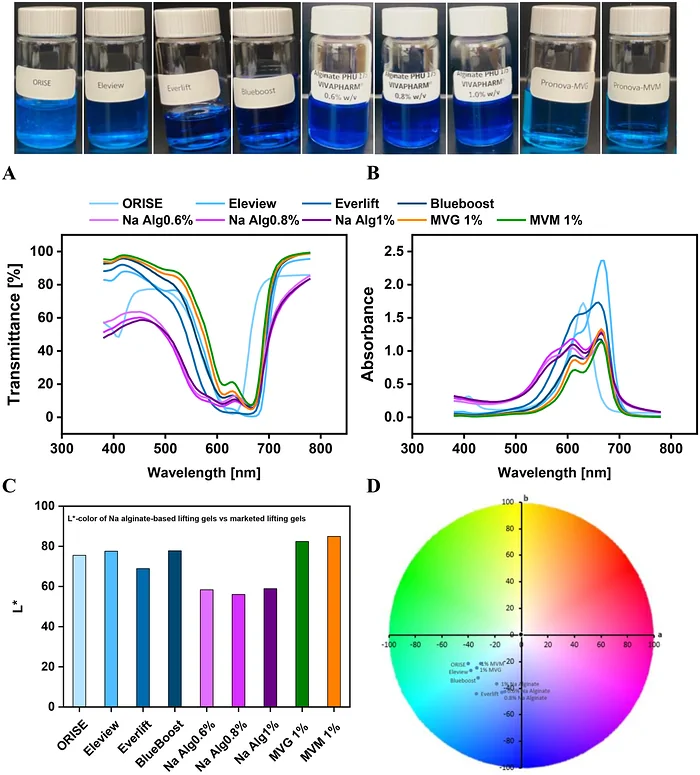
Color evaluation of JRS Na Alg, NovaMatrix MVG and NovaMatrix MVM‐based lifting gels in comparison to marketed lifting gels, namely, ORISE Gel, Eleview, EveLift and BlueBoost using UV visible spectrophotometer by CIE lab model. [A] % transmittance and [B] absorbance as evaluated by UV visible spectrophotometer at 380 nm to 780 nm wavelengths. Color evaluation by CIE L*a*b* space model [C] L* graph (related to the luminosity of the color) and [D] a* (defines the red or green component of the color) and b* (defines the yellow or blue component) graph, the blue circle represents the point (a*, b*) of the sample.

The vibrant blue color evaluation of our developed Na alginate‐based lifting gels including JRS Na Alg (0.6, 0.8 and 1%), NovaMatrix MVG 1% and NovaMarix MVM 1% with methylene blue in comparison to marketed lifting gels is shown in Figure 4 and Table S4. In a typical experiment, the transmittance or and/ the absorbance spectra in the visible region (380‐780 nm) of all the samples was measured, then the mathematical expressions were used in the most commonly used color spaces (CIE XYZ, CIE L*a*b*, CIE LCH, and RGB) to obtain the color coordinates, specifically, for color comparison. The color space analysis reveals notable differences in visual properties among the formulations. NovaMatrix MVG 1% and NovaMatrix MVM 1% exhibit the highest CIE XYZ values, indicating superior brightness and color intensity, while JRS Na Alg 0.8% and 1% show the lowest, suggesting reduced luminance. In the CIE Lab model, lightness varies significantly, with NovaMatrix MVM 1% being the brightest and JRS Na Alg 0.8% the darkest. All samples show negative a and b values, reflecting a shift toward green and blue hues, with JRS Na Alg variants appearing more blue‐shifted. EverLift demonstrates the highest chroma in the CIE LCH space, indicating strong saturation, and all formulations fall within the blue‐green hue range. RGB data further confirms the dominance of green and blue channels, especially in NovaMatrix MVG and NovaMatrix MVM, while EverLift and BlueBoost show the most intense blue values. Overall, these findings suggest that NovaMatrix MVG and NovaMatrix MVM formulations offer enhanced visual clarity, making them ideal for applications requiring high contrast and color distinction.

The perceptual color difference between samples can be quantified using the Delta E (ΔE) metric defined by the following equation (see Equation 1): [56]

(1) $$\Delta E_{ab}^{*}=\sqrt{{\left(L_{2}^{*}-L_{1}^{*}\right)}^{2}}+{\left(a_{2}^{*}-a_{1}^{*}\right)}^{2}+{\left(b_{2}^{*}-b_{1}^{*}\right)}^{2}$$

Delta E quantifies the difference between two colors based on their CIE LAB values. A ΔE less than 1.0 is generally considered imperceptible to the human eye. Values between 1.0 and 3.5 indicate a color difference that is noticeable to the human eye, ranging from barely perceptible to clearly visible. When ΔE exceeds 5.0, the difference is strong. The ΔE matrix comparing all formulations is shown in Figure S1. The ΔE matrix revealed that Eleview and Blueboost had a relatively low ΔE (7.2), suggesting similar color rendering. NovaMatrix MVG 1% and NovaMatrix MVM 1% showed a slight difference (ΔE = 4.9), likely due to identical dye type and concentration. These two formulations differed perceptibly from commercial products like ORISE Gel, Eleview, and Blueboost, with ΔE values ranging from 5.7 to 12.3. While other JRS Na Alg variants (0.6%, 0.8% and 1%) showed the highest ΔE values (27.3–36.3), indicating strong color shifts. EverLift had the most distinct color profile, with ΔE values exceeding 22 when compared to most formulations. Our gel formulations exhibited a comparable blue color to marketed products, with a clearer solution. Overall, our gels demonstrated consistent and aesthetically appealing coloration, with NovaMatrix MVG 1% and NovaMatrix MVM 1% standing out as promising candidates for further development.

### 2.6 Injectability of Lifting Gel

**FIGURE 5:**
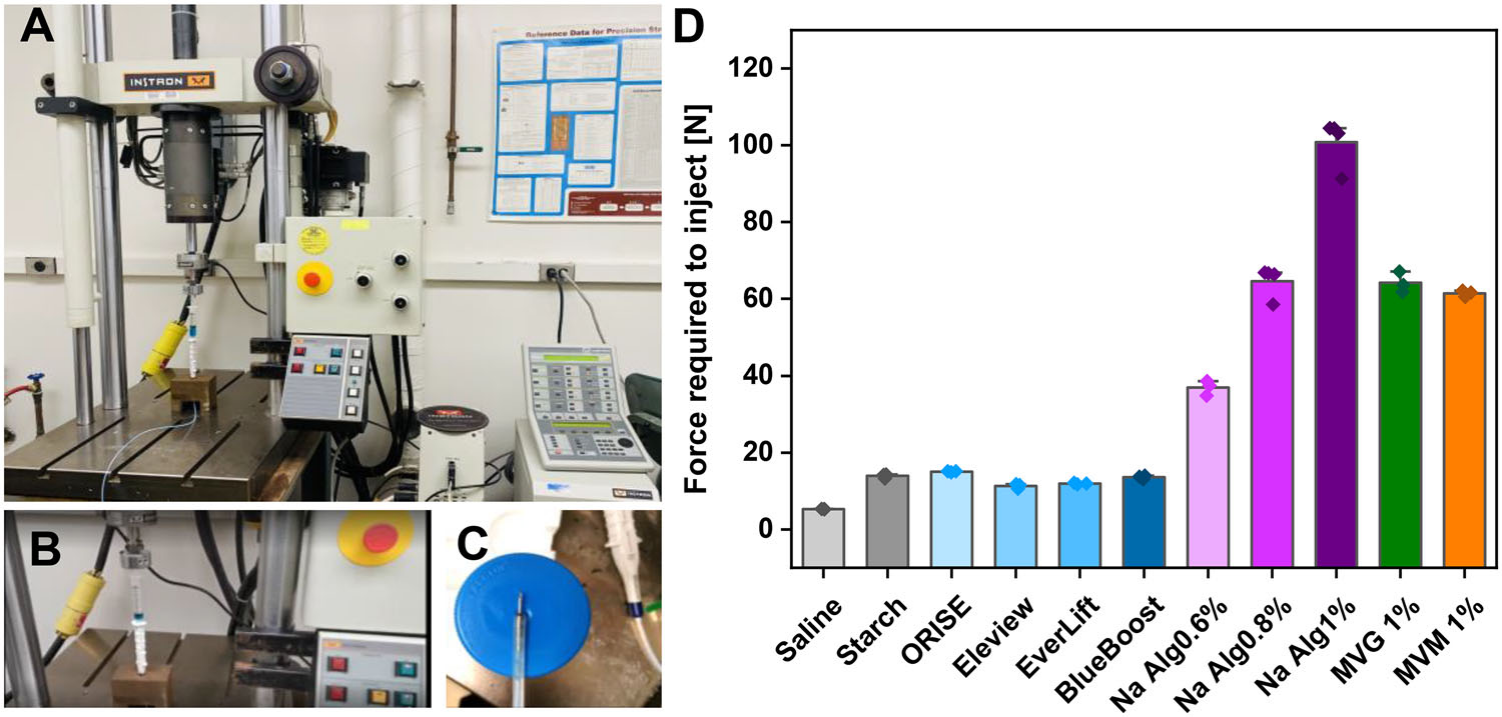
Injectability of lifting gels is measured using an Instron apparatus. [A] Instron apparatus with lifting gel [B] in 10 mL syringe in sample holder [C] through a 25 G endoscopy injection needle. [D] The mean force required to inject the Na alginate‐based lifting gels through a 25 G injection needle at various concentrations in comparison to commercially available products (saline, starch, ORISE Gel, Eleview, EverLift, BlueBoost). Columns with error bars represent means ± standard deviations (*n* = 3–4).

The mean force required to inject 10 mL of a gel solution through a 25 G endoscopy injection needle was measured using an Instron apparatus (see Figure 5). The force required to inject saline, starch, ORISE Gel, Eleview, EverLift, BlueBoost, JRS Na Alg 0.6%, JRS Na Alg 0.8%, JRS Na Alg 1%, NovaMatrix MVG 1% and NovaMatrix MVM 1% was: 5.28 ± 0.04, 13.96 ± 0.5, 15.05 ± 0.16, 11.31 ± 0.64, 11.94 ± 0.2, 13.60 ± 0.5, 36.98 ± 1.95, 64.57 ± 4.05, 100.8 ± 6.38, 64.14 ± 2.69 and 61.38 ± 0.82, respectively (see Figure 5D). The force required to inject various concentrations of Na alginate was significantly higher than the other commercial agents. Although this high viscosity challenges manual syringing, a mechanical screw‐assisted (spiral) syringe overcomes this limitation by providing stable, controlled delivery through a standard 25G needle during advanced endoscopic resections (ESD/EMR). Thus, the increased injection force does not limit the clinical utility of sodium alginate‐based lifting gel as a submucosal lifting agent.

The device package comprises a vial of lyophilized sodium alginate lifting gel and a screw‐assisted syringe. After reconstitution, the gel is injected using the screw mechanism, which provides mechanical advantage and allows smooth, controlled delivery while reducing the manual force required for viscous solutions in EMR and ESD procedures.

### 2.7 Stability Study

To study the effect the M to G ratio has on stability, both NovaMatrix MVG and NovaMatrix MVM were lyophilized in PBS and reconstituted with water; the samples were stored under accelerated conditions (40°C / 75% RH). For each timepoint the lyophilized cake was removed from stability chamber, inspected for visual appearance and reconstituted by hand shaking to measure reconstitution time, followed by gel appearance, pH, viscosity and osmolality measurement; the results are shown for NovaMatrix MVG and NovaMatrix MVM in Figure S2, and summarized in Table 7. Viscosity profiles were analyzed at 25°C (reconstitution and use temperature) and 37°C (physiological temperature) for each timepoint, revealing stable trends as shown in Table 7. Viscosity at 25°C was marginally elevated in comparison to that at 37°C representing temperature dependent change in viscosity. Conclusively, NovaMatrix MVG 1% w/v (sodium alginate) exhibited slightly higher viscosity than NovaMatrix MVM 1% w/v (sodium alginate) at the same concentration however, the overall viscosity patterns exhibited stability over the course of the study. Furthermore, no statistically significant alterations were detected in other critical parameters, including reconstitution time (60‐90 s), color (clear, blue), and pH (6.5) at the designated timepoints (t_0_: initial, t_1_: 1 month, t_6_: 6 months) as shown in Table 7. Furthermore, the osmolality (265–275 mOsm/kg) exhibited remarkable consistency across all timepoints, thereby indicating that the gels preserved their inherent characteristics throughout the storage interval.

**TABLE 7:** Lyophilized cake visual appearance, reconstitution time, color (appearance), pH and osmolality as well as Viscosity profile comparison at 25°C and 37°C at t0 (initial), t1 (1 month) and t6 (6 months) of NovaMatrix MVG 1% and NovaMatrix MVM 1% in PBS (0.8X, pH 6.5) Lifting gel stored under 40°C/75% RH.

	t_0_ (initial)		t_1_ = 1 month		t_6_ = 6 months	
NovaMatrix MVG 1% in PBS (0.8X, pH 6.5)						
Lyophilized cake appearance	Intact, uniform and elegant light blue cake		Intact, uniform and elegant light blue cake		Intact, uniform and elegant light blue cake	
Reconstitution time [S]	76.7 ± 6.24		67.3 ± 1.70		68.3 ± 2.49	
Color (appearance)	Clear, blue		Clear, blue		Clear, blue	
pH	6.50 ± 0.005		6.50 ± 0.005		6.49 ± 0.009	
Osmolality [mOsm/kg]	271.7 ± 1.70		269.3 ± 0.94			
	**25°C**	**37°C**	**25°C**	**37°C**	**25°C**	**37°C**
^1^Viscosity ± S.D. [mPa∙s]	58.2 ± 0.8	39.9 ± 1.1	57.1 ± 0.8	43.8 ± 0.4	59.4 ± 0.6	51.8 ± 2.5
^2^Viscosity ± S.D. [mPa∙s]	86.1 ± 0.9	54 ± 1.2	85.5 ± 1.3	59.8 ± 1.1	87.8 ± 0.8	75.1 ± 4.7
NovaMatrix MVM 1% in PBS (0.8X, pH 6.5)						
Lyophilized cake appearance	Intact, uniform and elegant light blue cake		Intact, uniform and elegant light blue cake		Intact, uniform and elegant light blue cake	
Reconstitution time [Seconds]	78 ± 6.68		70 ± 3.74		71 ± 4.32	
Color (appearance)	Clear, blue		Clear, blue		Clear, blue	
pH	6.50 ± 0.005		6.49 ± 0.009		6.49 ± 0.005	
Osmolality [mOsm/kg]	282.3 ± 2.05		282 ± 1.41			
	**25°C**	**37°C**	**25°C**	**37°C**	**25°C**	**37°C**
^a)^Viscosity ± S.D. [mPa∙s]	53.9 ± 3.9	43.8 ± 5.1	49.1 ± 0.5	41.2 ± 1.6	52.4 ± 1.1	43.4 ± 0.8
^b)^Viscosity ± S.D. [mPa∙s]	79.7 ± 3.8	61.9 ± 8.9	73 ± 1.0	56.8 ± 2.5	76.8 ± 2.2	61.5 ± 1.8

^a)^ Viscosity as measured at 1000 s^−1^ shear rate.

^b)^ Viscosity as measured at 142 s^−1^ shear rate.

### 2.8 Ex Vivo Lifting Hydrogel Performance

Ex vivo lifting of hydrogels were tested on porcine stomach specimens which have a close resemblance to human gastric tissue [57]. For the ex vivo studies, JRS Na Alg 0.6%, 0.8%, and 1%, NovaMatrix MVG 1% and NovaMatrix MVM 1% were compared with commercially available lifting agents and saline and starch were used as controls. Taking into consideration the results of mean force required to inject our developed Na alginate‐based lifting gel, we designed a spiral syringe with a handle which has the ability to turn and push the syringe in order to deliver viscous solutions. We conclude that with this specifically designed spiral syringe, even 1% w/v Na alginate solution could be delivered to the targeted area easily and accurately.

**FIGURE 6:**
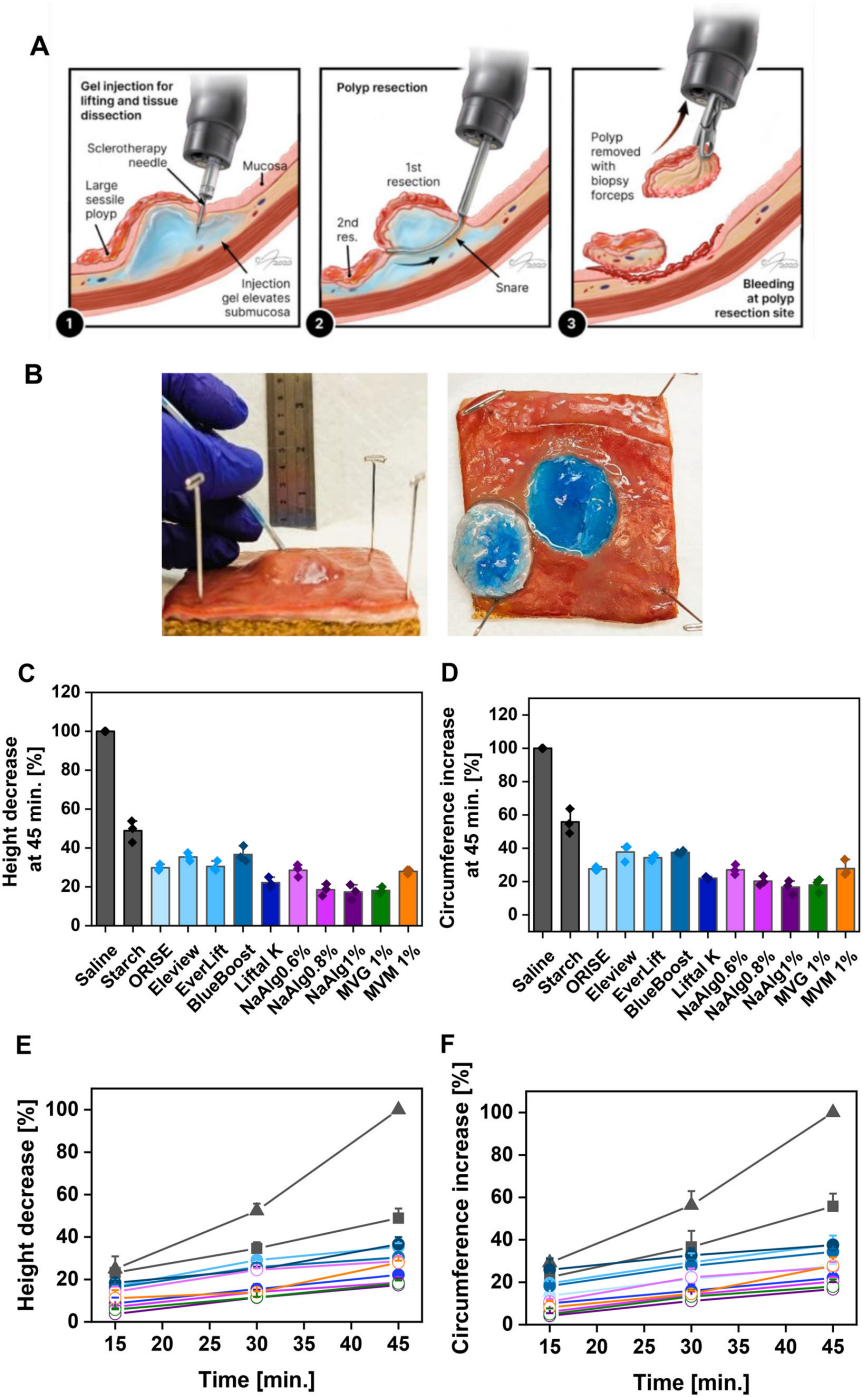
[A] Schematic representation of the application of injectable gel during the procedure of polyp excision utilizing the EMR methodology [1]. Diagrammatic depiction illustrates the administration of a lifting gel solution during the process of colon polyp excision with endoscopic intervention to facilitate the separation of gastrointestinal wall layers [2]. Excision of the polyp employing an endoscopic snare, frequently in conjunction with electrocautery [3]. Occurrence of hemorrhage subsequent to the lifting maneuver and polyp excision. Evaluation of the feasibility of injection of lifting gel using an endoscopy injection needle in the porcine gastrointestinal tissue. [B] Lifting gel depots are created, and cushion height is measured (left image). Following dissection presence of an intact submucosal depot is noted (right image). Ex vivo experiments evaluating the presence of submucosal cushions. Graphs demonstrate the percentage difference in [C] height and [D] circumference of the depot, 45 min after injection of the agent. This demonstrates the greater efficacy of the Na alginate‐based lifting gel in maintaining a stable depot in comparison to available marketed products. [E] The cushion height decrease percent and [F] cushion circumference increase percent of 7 Saline, Starch, ORISE Gel, Eleview, EverLift, BlueBoost, Liftal K, JRS Na Alg 0.6%, JRS Na Alg 0.8%, JRS Na Alg 1%, NovaMatrix MVG 1% and NovaMatrix MVM 1% after 15, 30 and 45 min. Columns and data points with error bars represent means ± standard deviations (*n* = 3–4).

Figure S5 illustrates testing for the lifting gel studies. Each agent was tested in triplicate, and the results were expressed as the mean and standard deviation of the cushion height decrease and circumference increase (see Figure 6, Figure S6 and Video S1). In this test, the JRS Na Alg 0.6%, 0.8%, and 1% and NovaMatrix MVG 1% and NovaMatrix MVM 1% showed a slower decrease in height and increase in circumference as compared to commercially available lifting agents, which is an advantage when conducting ESD procedures. The graphs (see Figure 6E,F) plot the height decrease and circumference increase vs time at 0, 15, 30, and 45 min; this data is derived from the studies shown in Figure S6.

In summary, the average percentage decrease in height of the JRS Na Alg: 0.6%, 0.8% and 1% was 14.2%, 7.0%, and 3.7% after 15 min and 28.6%, 18.5% and 17.3% after 45 min, respectively (Figure 6E). For NovaMatrix MVG 1% and NovaMatrix MVM 1%, we found the mean decrease in cushion height to be 5.8% and 11.1% after 15 min and 18.1% and 27.9% after 45 min, respectively. This was lower than the average percentage decrease in height seen for the controls and commercially available agents: saline, starch, ORISE Gel, Eleview, EverLift, BlueBoost and Liftal K was 24.9%, 23.2%, 15.9%, 16.7%, 16.2%, 18.4% and 8.8% after 15 min while 100%, 48.9%, 29.9%, 35.4%, 30.4%, 36.6% and 22.1% after 45 min, respectively (Figure 6C–F). A similar trend was seen with the average percentage increase in circumference during these timepoints (Figure 6F). Further, the JRS Na Alg 1.2% (data not shown) was tested and had an even slower height decrease (8.3% after 45 min) and circumference increase (7% after 45 min) than the JRS Na Alg 1%, and 0.8% concentrations. However, the nature of the lift was too hard to perform an ESD, or EMR procedure; thus, making it undesirable to use as a lifting agent; thus, we confined our studies to JRS Na Alg concentrations less than 1.2%.

### 2.9 Efficacy Testing in the Intact Stomach

The lifting hydrogel was subjected to evaluation utilizing an ex vivo intact stomach model to investigate its injectability, lift persistency, and graspability with snare across the antrum, body, and fundus (see Figure S7 and Video S2). NovaMatrix MVG 1% and JRS Na Alg 0.8% exhibited good injectability across all anatomical regions, necessitating minimal force application and yielding well‐defined, enduring mucosal elevations (Table S6). These findings endorse NovaMatrix MVG 1% as an optimal concentration that effectively balances viscosity and stability. Endoscopic handling assessments revealed that the lifts produced using JRS Na Alg 1% were readily graspable with a cold snare, notwithstanding the gel's higher viscosity, with no observed slippage or recoil, thus indicating its compatibility with endoscopic mucosal resection (EMR) procedures. Collectively, the gels yielded consistent, stable, and clinically pertinent lifts, substantiating their application as efficacious submucosal lifting agents within an ex vivo gastric model.

### 2.10 In Vivo Evaluation of Injectable Gel

**FIGURE 7:**
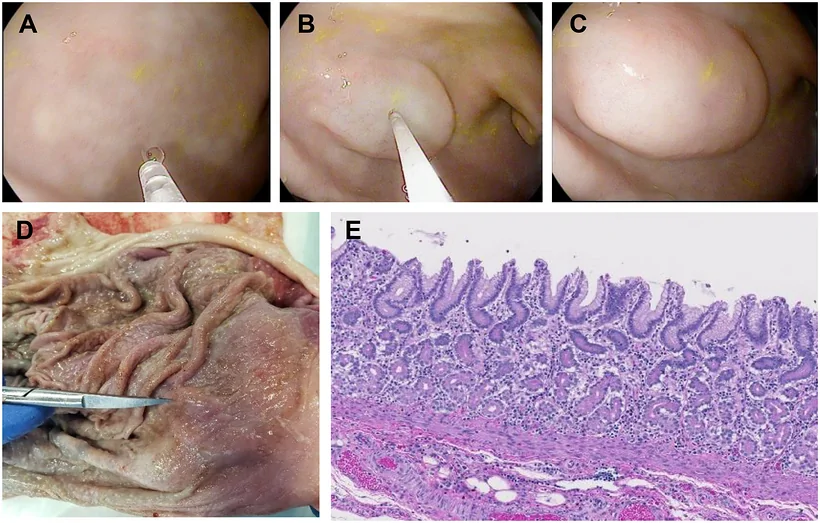
An in vivo experiment with injection of Na alginate‐based lifting gels and commercial gel into a pig stomach using endoscope. [A] and [B] in vivo experiments with injection of Na alginate‐based lifting gel into a pig stomach endoscopically. [C] Endoscopy view of stomach after gel injection [D] Autopsy examination of gel depot in pig stomach after 3 days. [E] Microscopic examination at the injection site in the stomach demonstrating no adverse reaction.

In vivo studies were conducted in live pig stomach and depots of up to 40 mL hydrogels were successfully created in the gastric submucosa as demonstrated in Figure 7A–C. Direct endoscopic visualization confirmed the presence of a submucosal cushion at 15 min with all the formulations tested. At 45 min, only the Na alginate ‐based lifting gel formulations and commercial gel (ORISE Gel), showed the presence of submucosal cushion. The results are summarized in Table S7. This confirmed that the Na alginate ‐based lifting gel formulations were better at submucosal lifting compared to the controls and some commercial gels.

#### 2.10.1 Safety Studies

In order to evaluate the safety profile of the in house developed gel formulations, euthanasia of the animal subjects was conducted, followed by comprehensive necropsy examinations. All porcine subjects successfully completed the designated survival durations (days 3 and 14) without experiencing any complications. At day 3, all specimens exhibited a consistent submucosal elevation (median dimensions of 5.5 × 5.5 cm). One site exhibited mild hyperemia; however, all remaining sites appeared unremarkable, devoid of any discoloration or tissue damage. The lesions were characterized by the presence of either blood–fluid mixtures or pale blue‐gray amorphous substances. No adverse events were reported (see Table S8, Figure 7D). Histopathological analysis indicated that the mucosal layer remained intact, with only minimal evidence of inflammation (see Figure 7E). At day 14, macroscopic examination revealed the absence of discoloration or tissue degradation, while one site sustained a persistent elevation measuring 4.5 × 3.0 cm. Histological assessment revealed mild submucosal edema and slight leukocyte infiltration in one specimen, whereas all other sites displayed only negligible inflammation. The architectural integrity of the mucosa was preserved, thereby supporting the safety profile of the sodium alginate lifting gels. Also, partial resorption of the gel was noted (Table S8).

It is important to highlight here that we extensively assessed the performance of novel Na alginate‐based submucosal injectates for ESD/EMR compared to existing agents. Although alginate‐derived lifting agents such as Liftal‐K are presently accessible, our formulation is presented in a lyophilized (freeze‐dried) form that can be readily reconstituted prior to application. This methodology markedly enhances product stability and longevity in comparison to Na alginate‐based liquid formulations, as corroborated by our internal stability assessments. Furthermore, the elevated viscosity of our Na alginate‐based lifting gel formulation yielded excellent ex vivo and in vivo submucosal cushioning efficacy and longevity relative to commercially available alternatives, thereby indicating augmented functional and practical benefits.

The novelty of our Na‐alginate lifting gel formulation lies not only in its higher viscosity but also in its lyophilized (freeze‐dried) form for ease of reconstitution and enhanced stability compared to comparable liquid alginate formulations. Also, preliminary results of our Na Alg‐based lifting gel demonstrated efficacy in underwater EMR/ESD applications by improving buoyancy, visualization, reducing bubble formation, and minimizing thermal injury, proving particularly effective for large, flat, or fibrotic lesions.

While EMR and ESD are established for treating superficial GI lesions, ESD offers superior en bloc resection, especially for larger lesions [22, 58]. A durable submucosal cushion is critical for procedural safety and efficiency [59]. Ideal injectates should provide prolonged tissue elevation to minimize perforation risk and reduce reinjection frequency [60]. Although ESGE recommends viscous solutions over saline for EMR, specific guidelines for ESD injectates are lacking, and most are currently used off label [61]. Our formulation is designed to promote safe submucosal lifting in gastrointestinal endoscopic procedures. Its enduring cushioning aids both routine and complex interventions needing multiple applications. Notable attributes include extended elevation, substantial tissue expansion with minimal volume, enhanced viscosity for accuracy, and total resorption over time. These aspects improve its utility in various endoscopic resection procedures, particularly for larger, intricate lesions which are typically managed with ESD rather than EMR.

### 2.11 PCA Modeling of Data

In order to evaluate how the variation in physical characteristics of the lifting gel for example viscosity and ‘n’ (the flow behavior index) has an overall effect on lifting persistency of lifting hydrogels or in simple words, the injectability, the percent decrease in lift height after a duration of 15‐, 30‐ and 45‐min, the increase in lift circumference after the same period, the injectability, the graspability of the lift with snare (feel), and the persistence of lifting (Lift) (see Table S9). A PCA model was developed using the above‐mentioned autoscaled process variables for each of the following formulations saline, starch, ORISE Gel, Eleview, EverLift, BlueBoost, Liftal K, JRS Na Alg 0.6%, JRS Na Alg 0.8%, JRS Na Alg 1%, NovaMatrix MVG 1% and NovaMatrix MVM 1%. A total of three principal components (PCs) were identified as significant, collectively accounting for approximately 98.57% of the overall variance observed (see Figure S4).

**FIGURE 8:**
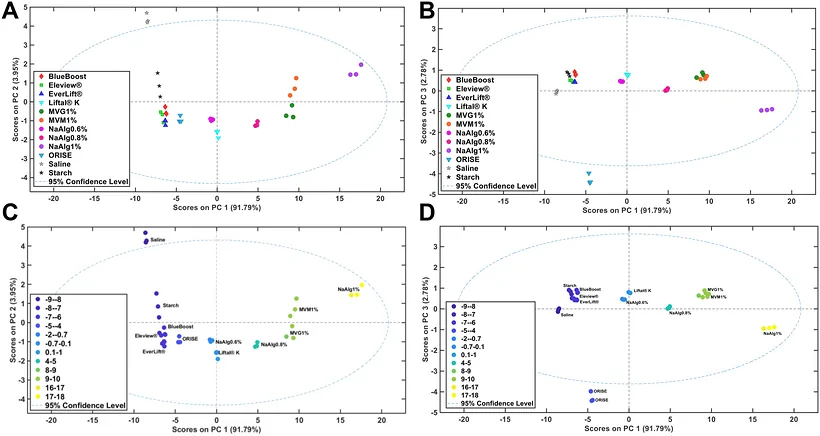
Principal Component Analysis (PCA) models utilizing the viscosity profiles of representative hydrogels, in conjunction with the variables of viscosity index (n), together with ease of injectability of formulation in tissue, percent lift height decrease, breadth increase as well as the graspability of the lift with snare. [A] the PC1 vs. PC2 score plot, [B] the PC1 vs. PC3 plot. [C] and [D] The corresponding heatmap visually represents the relationships across a gradient of physicochemical and functional attributes, ranging from blue (poor) to green (optimum/ideal) to yellow (undesirable or excessively viscous) of the PC1 vs. PC2 and PC1 vs. PC3 score plots, respectively.

The PC1 vs. PC2 score plot (see Figure 8A) revealed distinct clustering of JRS Na Alg 0.8% and NovaMatrix MVG 1% in the lower right quadrant, clearly separated from other formulations. This clustering pattern indicates their consistent and advantageous physicochemical and functional properties. In contrast, the control samples (saline and starch) appeared in the upper left quadrant of the PCA space, reflecting their low viscosity and poor lift quality. The commercial formulations (ORISE Gel, BlueBoost, EverLift, and Eleview) occupied separate quadrants, highlighting pronounced differences in their rheological and functional characteristics compared to the alginate‐based formulations. The corresponding heatmap (see Figure 8C) visually represents these relationships across a gradient of physicochemical and functional attributes, ranging from blue (poor) to green (optimum) to yellow (undesirable or excessively viscous). Formulations highlighted in green namely JRS Na Alg 0.8%, NovaMatrix MVG 1%, and NovaMatrix MVM 1% exhibited the most desirable balance of viscosity and lift performance among all tested samples. Similarly, in the PC1 vs. PC3 plot (see Figure 8B), the in‐house developed Na‐alginate–based lifting gels demonstrated favorable shear‐thinning behavior coupled with superior lift persistence compared to the marketed formulations. ORISE Gel, however, appeared as an outlier beyond the 95% confidence ellipse, likely due to its exceptionally high initial viscosity and pronounced shear‐thinning behavior upon deformation. The heatmap corresponding to the PC1 vs. PC3 model (see Figure 8D) further confirmed that formulations located in the upper and lower right quadrants (represented in green) possess optimal physicochemical (viscosity) and functional (lift persistency) properties. We also evaluated other measured physicochemical parameters as variables in the PCA model, however, adjusted osmolality (<300 mOsm/kg) and pH (6.0–7.5) of all injectable formulations showed no correlation with lift persistency. Therefore, the PCA results indicate that viscosity and ‘n’ (the flow behavior index) are the key parameters that dictate and strongly correlate with mucosal lift persistence. Also, a closer examination of the PC1 score plot (Figure S5) revealed a clear differentiation among the tested mucosal lifting gels, with all samples falling within the 95% confidence interval except for JRS Na Alg 1%, which showed a higher deviation likely due to its markedly high viscosity.

Altogether, both PCA model and correlation (Figure S6) revealed that viscosity and the flow behavior index (n) are critical rheological determinants governing mucosal lift persistency. In summary, formulations with higher viscosity and optimized shear‐thinning behavior exhibited superior lift stability and retention, emphasizing the importance of rheological tuning in the design of submucosal injection solutions.

In summary, our in vitro, ex vivo and in vivo investigations elucidate that sodium alginate‐based lifting gels produce a more enduring and stable submucosal cushion compared to traditional agents such as saline and starch, thereby diminishing the necessity for reinjection and enhancing procedural efficacy and precision. In contrast to ORISE Gel, these gels exhibit biocompatibility and do not impede histological evaluations, whilst still providing improved visualization through the use of premixed dye without excessive pigmentation. Coupled with ease of preparation and a reduced financial burden in comparison to proprietary agents, sodium alginate‐based gels signify a noteworthy progression in the domain of submucosal lifting technology.

## 3 Conclusions

Advances in submucosal lifting agents have significantly improved the safety and efficiency of EMR and ESD. From simple solutions like saline to sophisticated hybrid gels, these innovations are shaping the future of minimally invasive endoscopic therapy. Herein, we have engineered a sodium alginate‐based injectable hydrogel that provides a long‐lasting and stable submucosal elevation, enhanced visualization, and remarkable biocompatibility, thereby effectively addressing the primary shortcomings of currently available lifting agents. The assessment of endotoxin‐free materials led to the identification of NovaMatrix MVG 1% as the most advantageous formulation, exhibiting comparable injectability, reliable snare graspability, and consistently sufficient lifting capability in both ex vivo and in vivo assessments. In comparison to established agents including ORISE Gel, Eleview, EverLift, BlueBoost, saline, and starch, NovaMatrix MVG 1% exhibited improved handling characteristics and procedural efficacy. In summary, these results underscore the promise of this sodium alginate‐based hydrogel as a prospective next‐generation submucosal lifting agent for EMR and ESD.

This study focused on the development and optimization of a Na Alg‐based lifting gel, evaluating its in vitro, ex vivo, and in vivo performance metrics. Future research should entail thorough biocompatibility evaluations, comprising cytotoxicity and cell proliferation analyses. Ongoing preclinical and clinical investigations will be crucial to substantiate its efficacy in wider applications and to facilitate its incorporation into standard endoscopic practice for enhanced patient outcomes.

## 4 Experimental Section

### 4.1 Materials

Sodium alginate (PRONOVA UltraPure Alginates named as Pronova UP MVG/ Pronova UP MVM) was purchased from NovaMatrix (IFF N&H Norway AS)). Also, sodium alginate (VIVAPHARM Alginate PHU 175) was procured from JRS PHARMA (France). Phosphate buffered saline (PBS) (10X), pH 7.4 without Calcium & Magnesium was commercially available and from Quality Biological. ProvayBlue (methylene blue) Injection, USP 50 mg/10 mL was obtained from American Regent, Inc. (Shirley, NY, USA). We obtained Eleview (Cosmo Pharmaceutical Ltd. NV), EverLift (Laborie Medical Technologies corp.), BlueBoost (Microtech Endoscopy), ORISE Gel (Boston Scientific), saline (BD PosiFlush, NJ, USA) and starch (HEXTEND, Hospira Inc. IL, USA). Further details, including batch numbers or lot numbers of all the above‐mentioned chemicals and products are available in Tables S1 and S2.

### 4.2 Study Design

In this study, we developed a series of injectable endoscopically deliverable sodium alginate‐based gels as a lifting agent for mucosal and submucosal dissection to determine the best formulation properties for these endoscopic procedures. To this end, the sodium alginate raw materials were sourced from JRS Pharma and NovaMatrix with their respective acronyms detailed in Table 1 as JRS Na Alg and NovaMatrix MVG and NovaMatrix MVM. In detail, different concentrations of sodium alginate from different sources, for example, JRS Na Alg and NovaMatrix MVG and NovaMatrix MVM were optimized and assessed to determine the best concentration that kept a sustained lift (see Table 1). The detailed mechanical properties of the obtained hydrogels were examined by rheological tests. Further in vitro characterization included: pH, color, osmolality, syringeability, and compared with commercially available formulations. Ex vivo studies were designed in parallel to test the injectable hydrogel. The hydrogels with promising in vitro and ex vivo data were further tested in vivo. For large animal porcine studies, endoscopic submucosal injection was performed to evaluate for the feasibility of injection, evaluate the lifting, and characterize the performance in EMR, ESD procedures. All experiments were repeated three times unless otherwise mentioned (see Table 1).

### 4.3 Fabrication and Lyophilization of Sodium Alginate‐Based Lifting Gel

Sodium alginate of different concentrations, JRS Na Alg 0.6, 0.8 and 1% w/v and NovaMatrix MVG and NovaMatrix MVM 0.8 and 1% w/v were dissolved in phosphate buffered saline (PBS 0.8X) of pH 6.5 under magnetic stirring (400 rpm) for 18 h until a clear solution was formed. To the clear Na alginate solution, methylene blue (0.5 mg / 100 mL) was added to obtain blue colored lifting gel. In this way, the injectable sodium alginate‐based lifting hydrogels were formulated, and the gels were subsequently lyophilized for enhanced shelf‐life. In detail, we lyophilized 2 mL of sodium alginate‐based lifting gel solution (in buffer) in 5 mL lyophilzation vials using a three‐stage cycle. Samples were frozen by cooling from 0°C to −45°C (−1°C/min) and held for 180 min. Primary drying was performed at 100 mTorr with shelf temperature increased to −25°C and maintained until the Pirani–capacitance pressure difference (PRCM) was ≤5. Secondary drying was initiated at PRCM ≤5 by ramping to 25°C (0.2°C/min) and held until completion. Vials were capped under vacuum at the end of the cycle.

### 4.4 In Vitro Characterization of Lyophilized Lifting Hydrogels

The developed lyophilized injectable lifting hydrogel was reconstituted (in water or in buffer depending on the lyophilzation medium) by hand shaking prior to the procedure. It took us around 45 s to 1 min to completely dissolve the lyophilized cake to get a clear blue solution from the sky‐blue lyophilized cake. Upon reconstitution, the gels were characterized in vitro in detail including lyophilized cake appearance and color, reconstitution time, rheology, viscosity, pH, gel color, osmolality and gel injectability/syringeability, prior to ex vivo and in vivo studies and finally compared with commercially available formulations.

#### 4.4.1 Rheology of Lifting Gel

Viscosity assays were performed using a TA Instruments DHR‐2 Rheometer at 25°C and 37°C using a Cone and Plate (CP) of 1 degree and 40 mm diameter (40.0 mm 1°Cone and Plate, Peltier plate Stainless steel – 120301). Each experimental run consists of an “upcurve” where shear stress is measured as shear rate (rotational speed) is increased, followed by a “downcurve” where shear stress is measured as rotational speed is ramped down. The shear rate ranged from 0.01 to 10,000 s^−1^ for both the up and down curves. In addition, viscosity was also measured where the temperature was ramped up from 15°C to 45°C at a constant shear rate of 50 s^−1^ using a Cone and Plate of 1 degree and 40 mm diameter.

#### 4.4.2 Mathematical Assessment of the Rheological Behavior of Non‐Newtonian Fluids

The Power Law model is a mathematical representation of the rheological behavior of non‐Newtonian fluids. This model is predicated on the premise that the shear stress (σ) of a fluid is directly proportional to the shear rate ($\dot{\gamma }$) elevated to a specific exponent, referred to as the power‐law index (*n*). The mathematical formulation of the Power Law model, also known as the Ostwald de Waele relationship, can be articulated as follows (see Equation 2): [62]

(2) $$\sigma =K\cdot {\dot{\gamma }}^{n}$$

where σ (Pa or Dyne/ cm^2^) denotes the shear stress, *K* represents the consistency coefficient or the flow constant, $\dot{\gamma }$ (s^−1^) signifies the shear rate, and *n* indicates the power‐law index (the flow behavior index) [63]. The flow behavior index (n) for Newtonian fluids, n is equal to 1 (as the viscosity is independent of the shear stress or shear rate) for shear‐thinning fluids, 0< n < 1, and for dilatant or shear‐thickening fluids, 1< n < ∞. For shear thinning, the greater the deviation *n* has from 1 the greater is the shear thinning. The Power Law model is extensively employed to elucidate the behavior of pseudoplastic and dilatant fluids.

#### 4.4.3 PH and Osmolarity of Lifting Gel

The pH was measured using a pH meter (Thermofisher LSTAR1115) equipped with a Micro pH Electrode (Orion Thermofisher 9810BN) after performing calibration with pH 4.01, 7.00, and 10.01 buffers. While osmolality was measured using OSMETTE S Model 4002 Automatic Osmometer via freezing point depression osmometry. In a typical experiment, the osmometer was warmed up for 10 to 15 min after initial turn on to completely equilibrate the temperature. Then the osmometer was calibrated using the 500 mOsm standard with (mode switch to) “CAL I” first, and the 100 mOsm standard with (mode switch to) “CAL II” thereafter. Afterwards, the measuring sample was pipetted into clean, dry sample tubes and osmolality was determined by returning mode switch to “RUN” which measured the osmolality by freezing the samples. All experiments were performed in triplicate.

#### 4.4.4 Color Evaluation of Lifting Gel

For materials that reflect or scatter light, accurate color measurements require dedicated colorimetric analyzers capable of quantifying light scattering, specular and diffuse reflectance, and absorbance. Clear liquids have negligible scattering and reflectance, so color measurement can be done by absorbance as defined by the Beer‐Lambert law. Standard visible spectrophotometers can then be used to record the visible absorbance spectrum in the visible region (380‐780 nm). The spectrum is then transformed using the Commission Internationale de l'Éclairage (CIE) standard observer color matching functions, which model the spectral sensitivity of the human eye's cone photoreceptors. This transformation yields the CIE 1976 (L*, a*, b*) color space values, where L* represents lightness, a* denotes the red‐green axis, and b* corresponds to the yellow‐blue axis. These parameters quantitatively describe color as perceived by the human eye, allowing for comparison between samples [64, 65, 66].

Methodology including and Excel‐based calculator described by Delgado‐Gonzalez et al. [64] was used to calculate the CIE LAB color coordinate [64]. The blue color lifting gels were measured using a UV–vis Spectrophotometer (UV‐1600 PC Spectrophotometer, VWR) at 5 nm intervals using a 1 cm cuvette. The spectral data was input into the “Template‐5 nm‐for‐Researchers” workbook. The calculation used the CIE standard observer color matching functions to transform the spectra to the CIE LAB coordinates. The CIE LAB (L*a*b*) color space was used to compare the obtained blue color of lifting gels with marketed gels [65]. The formulas used in the Excel model are derived from CIE reports [65, 66]. The model was adapted to ISO Normative in 2008 (ISO 11664‐4:2008).

#### 4.4.5 Injectability of Lifting Gel

The force required to inject the selected gel through the endoscopy needles was measured using an Instron Model 8500 apparatus (Instron Corporation, USA) equipped with a 1000 N load cell operating in compression mode and Novell software for analytical data processing. A bespoke steel holder was employed to stabilize and hold the syringe normal to the compression load during testing. The experimental procedure was carried out at ambient temperature (25°C), with a crosshead velocity of 40 mm/min, thereby compressing the syringe plunger to a predefined distance (8 mL within a 10 mL syringe). Data corresponding to the initial 10 mm of displacement were omitted due to the influence of transient starting conditions. A total of three replicates were conducted for each gel sample. In a typical experiment, 10 mL of the test gel solution was injected through a 25 G injection needle, and the maximum pressure required to inject the solution was recorded. In brief, the gel was introduced into the syringe barrel, which was subsequently positioned within the holder. Data pertaining to the empty syringe were recorded to evaluate the injection force.

#### 4.4.6 Stability Study of Lyophilized Lifting Gel

This study examines a key physical instability with alginate gels, the loss of viscosity due to chain breakdown. There are two questions this study will examine: 1) does the mannuronic to guluronic ratio, affect the rate of chain breakdown and 2) how does the lyophilization and reconstitution buffer system influence gel stability. The stability of lyophilized sodium alginate‐based lifting gels was assessed using standard ICH storage conditions, real time (25°C / 60% RH), intermediate (30°C / 65% RH) and accelerated (40°C / 75% RH) for a duration of six months, with sampling timepoints at 0, 1 and 6 months, and the stability parameters assessed are changes in lyophilized cake appearance, reconstitution time, solution visual appearance, viscosity, pH and osmolality.

### 4.5 Ex Vivo Screening of Gel Formulations

The selected gel formulations were screened in ex vivo settings using porcine gastric tissue and endoscopic accessories replicating the environment during endoscopy.

#### 4.5.1 Cushioning Efficacy of Submucosal Lifting Agents

Porcine stomach tissue was used to determine the duration for which the gel formulations when injected into the submucosal space created adequate cushion lifting to facilitate endoscopic resection of a polyp. Fresh porcine stomachs were procured from a local grocery store, trimmed into 3 × 3‐inch specimens, and mounted on cork boards. During the course of experiment, the gastrointestinal specimens were maintained at 37°C using temperature‐controlled heating pads to mimic human body temperature. A total of 2 mL of gel was injected into the submucosal space using a 25G endoscopic injection needle (Boston Scientific, Marlborough, MA). The initial lift height (t_0_), along with the width across the major and minor axes, was measured using vernier calipers. Subsequent decrease in height and increase in circumference of the submucosal cushion were obtained at 15‐, 30‐, and 45‐min post‐injection considering 45 min as an average ESD procedure time. The experiment was performed in parallel with commercial gels, starch, and saline as controls, with each agent tested in triplicates. The duration of the submucosal lift was calculated as a cushion height decrease (%) using Equation (3).

(3) $$\mathrm{Cushion}\;\mathrm{height}\;\mathrm{decrease}\;\;\left[\%\right]=\frac{Ht_{0}-\;Ht_{i}}{Ht_{0}}\times 100$$

where Ht_0_ and Ht_*i*_ are the cushion height at t = 0, 15, 30, 45 min timepoints. In addition, an increase in the circumference of submucosal lift was simultaneously determined by measuring the length of major and minor axes of oval shaped lift with a vernier calipers and approximating the circumference of the lift using the following equation (Equation 4):

(4) $$\mathrm{Cicumference}\;\mathrm{of}\;\mathrm{oval}\;\mathrm{lift}=2\pi \sqrt{\frac{a^{2}+b^{2}}{2}}$$

where *a* is the major axis and *b* is the minor axis with π value taken as 3.14. These two equations were used to characterize the lift geometry and its change.

#### 4.5.2 Efficacy Testing of Lifting Agent in an Intact Stomach

An ex vivo model has been developed based on a porcine upper gastrointestinal model from Endosim (Endosim, item number SQ0026112, Upper GI Specimen, porcine stomach) where an intact stomach with esophagus is used to simulate the gastric environment. The gel (3–4 mL) was injected endoscopically into the submucosal space at three anatomical locations, namely, antrum, body, and fundus in order to assess the ease of injectability, lift persistency, and the graspability of the lift. Injectability was determined subjectively by scoring the force required to expel the gel through the syringe while lift persistency was assessed based on the sustainability of the lift observed endoscopically for 45 min and graspability of the lift by using a 15 mm cold snare.

### 4.6 In Vivo Animal Studies

A healthy swine model was selected for this study due to its gastrointestinal anatomy and size, which closely resembles those of humans, making it a clinically relevant species for evaluating gastrointestinal medical therapies [67, 68]. Adolescent male Yorkshire swine (∼50 kg) were purchased from Archer Farms and acclimatized for at least 7 days before undergoing procedures. The swine were housed (2 per cage) at the Animal Core Facility of Johns Hopkins University School of Medicine, under the oversight of the Office of Animal Welfare Assurance (OAWA), which adheres to guidelines mandated by the Institutional Animal Care and Use Committee (IACUC). All procedures were conducted in compliance with IACUC protocol #SW23M360 reviewed and approved by the Johns Hopkins University Animal Care and Use Committee (ACUC) on 1/2/2024. Animals were fasted for 24 h prior to surgery. For all procedures, animals were anesthetized by standard protocols. At the end of the study period that animals were euthanized using TKX (a cocktail made of 500 mg Telazol, 250 mg Ketamine (100 /mL), and 250 mg Xylazine (100 mg/mL)), administered IM.

#### 4.6.1 In Vivo Evaluation of Injectable Gel for Mucosal Lifting

The gel polymers which showed optimal cushion lift in ex vivo setting were selected for in vivo testing. In vivo studies were conducted in live pig stomach in order to evaluate the feasibility of delivering and efficacy of lifting of Na alginate‐ based lifting gels and commercially available lifting gels in comparison to controls through an endoscopic 25 G injection needle. As ex vivo studies showed that depots of 40 mL can be created, the same amount of gel was injected in the in vivo setting. In a typical experiment, the hydrogels were injected into a live pig stomach using an endoscope with a 25 G injection needle and depots of up to 40 mL were successfully created in the gastric submucosa. The performance of the gel polymers was compared with positive control (starch), negative control (saline) and commercial gels. The primary endpoint was confirmation of presence or non‐existence of a submucosal cushion under direct endoscopic visualization at 45 min.

#### 4.6.2 Safety Studies

The safety studies were performed to confirm that the proposed gels were safe to use in live animals by creating submucosal depots in porcine stomach tissue, followed by gross and microscopic assessment. In a typical set of experiments, six Yorkshire pigs were used and divided into two groups, with survival periods of 3 and 14 days, respectively. In each animal, 10 mL of gel was injected into the submucosal space using a 25G needle and the site of injection was marked with dye. At the designated timepoints (day 3 and day 14), necropsy was performed and the injection sites were examined for gross and microscopic changes. Gross analysis involved subjective assessment of discoloration and tissue disintegration, scored on a 1–4 scale. Following gross evaluation, stomach specimens were formalin‐fixed for 24 h, processed for histology, digitized, and reviewed by a pathologist. Histological evaluation included severity of inflammation, mucosal vitality, architectural changes, and hematomas. Inflammation and fibrosis were scored on a four‐point scale (none, mild, moderate, severe).

### 4.7 Principal Component Analysis (PCA) Modeling

Principal Component Analysis (PCA) is a powerful method for exploring the relationship between multiple samples and variables [69]. To assess the collective impact of physical and performance‐related characteristics on the injectability and lifting persistency of hydrogel formulations, PCA model was employed. The variables included viscosity, flow behavior index (n), percent decrease in lift height at 15, 30, and 45 min, increase in lift circumference over the same time intervals, graspability with snare (feel), and persistence of lift. Given the diverse measurement scales and magnitudes of these parameters, autoscaling was applied prior to PCA. The principal components were identified to capture the greatest sources of variance in the data and facilitate interpretation of relationships among formulation properties and lift performance.

### 4.8 Statistical Analysis

The statistical analysis was performed using OriginPro 2019b (OriginLab Corporation, Northampton, MA, USA). Unless otherwise stated, all the experiments were repeated at least three times and results were represented as means ± standard deviations. One‐way, two‐way, and three‐way analysis of variance (ANOVA) followed by Tukey's Post Hoc test was performed in order to find statistically significant results (*p* ˂ 0.05) within the datasets. Then data analysis was performed in order to correlate the best set of physical and chemical properties, e.g., viscosity for lifting gel performance using MATLAB R2024b (The MathWorks, Inc., Natick, MA, USA) and PLS toolbox 9.5 (R9.5) (Eigenvector Research, Inc., Wenatchee, WA, USA).

## Author Contributions

Iram Maqsood: Conceptualization, Methodology, Software, Validation, Formal analysis, Investigation, Data curation, Visualization, Writing –original draft, Writing –review & editing. Venkata S. Akshintala: Conceptualization, Resources, Writing –review & editing, Supervision, Project administration, Funding acquisition. Surya Evani: Methodology, Investigation, Writing –original draft. Rebecca A. Krimins: Investigation. Yihan Wang: Investigation. Keith Freel: Investigation, Software. Ahmed Ibrahim: Software. Saowanee Ngamruengphong: Investigation. Mouen Khashab: Investigation. Stephen W. Hoag: Conceptualization, Methodology, Validation, Resources, Writing –review & editing, Supervision, Project administration, Funding acquisition.

## Funding

We acknowledge Origin Endoscopy and Maryland Innovation Initiative (MII) TEDCO (Grant No. 204363) for funding this project.

## Conflicts of Interest

VSA and SWH are Co‐founders of Origin Endoscopy LLC, Company developing endoscopic gels.

## Supporting information




**Supporting File 1**: adhm71427‐sup‐0001‐SuppMat.docx.

**Supporting File 2**: adhm71427‐sup‐0002‐VideoS1.mp4.

**Supporting File 3**: adhm71427‐sup‐0003‐VideoS2.mp4.

## Data Availability

The data that support the findings of this study are available from the corresponding author upon reasonable request.
