# Supplementary material for: Sodium Alginate‐Based Submucosal Lifting Hydrogel for Endoscopic Mucosal Resection and Endoscopic Submucosal Dissection
Source: Adv Healthc Mater. 2026 Jul 11;15(32):e71427. doi: 10.1002/adhm.71427 (PMC13507562; doi:10.1002/adhm.71427)
Supplement: Supplementary file 1 [file ADHM-15-0-s003.docx]

# Supporting Information

Sodium Alginate-based Submucosal Lifting Hydrogel for Endoscopic Mucosal Resection and Endoscopic Submucosal Dissection

*Iram Maqsood^1 #^*, Venkata S. Akshintala^2 #^, Surya Evani^2^, Rebecca A. Krimins^3^, Yihan Wang^1^, Keith Freel^1^, Ahmed Ibrahim^1^, Saowanee Ngamruengphong^2^, Mouen Khashab^2^ and Stephen W. Hoag^1^**

**Affiliations**

^1^Department of Pharmaceutical Sciences, University of Maryland School of Pharmacy, 20 N Pine Street, Baltimore, MD 21201, USA

^2^Division of Gastroenterology, Johns Hopkins Hospital, Baltimore, MD 21287, USA

^3^Express Radiology Research Lab, Johns Hopkins Hospital, Baltimore, Maryland

***Correspondence:

**Iram Maqsood, Ph.D.**

School of Pharmacy, University of Maryland, Baltimore, MD 21201, USA

**Email:** imaqsood@rx.umaryland.edu

**Phone: +1(443)794-3120**

**Stephen W. Hoag, Ph.D.**

School of Pharmacy, University of Maryland, Baltimore, MD 21201, USA

**Email:** shoag@rx.umaryland.edu

**Phone: 410-706-6865**

^#^contributed equally to the first authorship

**This file includes additional Results for Article.**

- **Table S1:** Composition and source of our lead injectable mucosal lifting gel.
- **Table S2:** Source of commercially available lifting agents used for comparative evaluation.
- **Table S3:** Typical ingredients in a mucosal lifting gel.
- **Table S4:** Colorimetric assessments of gels across various color spaces.
- **Figure S1:** Similarity heatmap produced from the ΔE matrix comparing color quantification across all formulations.
- **Figure S2:** Stability study of lyophilized NovaMatrix MVG 1% and NovaMatrix MVM 1% lifting gel in PBS when stored under accelerated condition (40 °C/75%RH) at t_0_ (initial), t_1_ (1 month) and t_6_ (6 months).
- **Table S5:** Stability study of NovaMatrix MVG 1%-based lifting gel lyophilized in PBS or lyophilized in water and reconstituted in water or PBS respectively.
- **Figure S3:** Stability study of NovaMatrix MVG 1% lifting gel lyophilized in PBS when stored under regular (25 °C / 60% RH) and intermediate (30 °C / 65% RH) conditions at t_0_ (initial), t_1_ (1 month) and t_6_ (6 months).
- **Figure S4:** Stability study of NovaMatrix MVG 1% lifting gel lyophilized in water and reconstituted in PBS stored under [A] 25 °C/60% RH and [B] 30 °C/65% RH at t_0_ (initial), t_1_ (1 month) and t_6_ (6 months).
- **Figure S5:** Schematic illustration of assessing the cushioning efficacy of lifting agent.
- **Figure S6:** The cushioning efficacy of lifting agent and lifting persistence assessment in the porcine model.
- **Figure S7:** Graphical representation of injection of lyophilized-reconstituted lifting gel to the three different locations of stomach, namely, fundus, body and antrum
- **Table S6:** The injectability, graspability with snare and lift of lifting gels in the ex vivo intact stomach model
- **Table S7:** In vivo evaluation of submucosal cushion for at least 45 minutes when injected into live porcine GI tissue.
- **Table S8:** Gross and histological changes at the injection site following necropsy in day 3 and day 14 survival groups.
- **Table S9:** Ex vivo performance of the lifting gels in porcine tissue.
- **Figure S8:** Loadings of the PCA model.
- **Figure S9:** PC1 score showing distribution of lifting gel within 95% confidence interval.
- **Figure S10:** Correlation between viscosity and mucosal lift parameters for various formulations.
- **Video 1:** Ex vivo mucosal lifting on porcine stomach specimens.
- **Video 2:** Mucosal lifting in intact stomach.

Supplementary tables and figures provide data on typical ingredients in a mucosal lifting gel, ingredient sources, and comparative ex vivo results of mucosal lifting using the sodium alginate-based gel and commercially available lifting agents. In detail, herein we have described the standard ingredients incorporated in mucosal lifting gels, alongside the precise formulation and sourcing details of our newly developed sodium alginate-based lifting gels. Additionally, it enumerates the commercial lifting agents employed for comparative analysis. The stability study of lyophilized NovaMatrix MVG 1% gel in PBS (0.8X, pH 6.5) when stored under accelerated condition (40 °C/75%RH), regular (25 °C / 60% RH) and intermediate (30 °C / 65% RH) conditions have described. Further, here we delineate the framework of ex vivo experiments utilizing porcine tissue to evaluate injectability, graspability with snare, and lifting longevity. Illustrative figures depict the performance of lifting over temporal intervals and the methodology adopted to assess the efficacy of cushioning, thereby substantiating the enhanced attributes of the alginate-based gel in comparison with commercially available agents.

**Table S1**: The composition and source of our sodium alginate-based injectable lifting gel are given below:

| **Ingredient** | | **Source** |
| --- | --- | --- |
|  |  |  |
| Sodium alginate | Pronova UP MVG | NovaMatrix  Batch Number: BP-2110-24  Specification Number: SPEC P 014  Product no.: 4200101, 4200106, 4200121 |
|  | Pronova UP MVM | NovaMatrix  Batch Number: BP-2104-13  Specification Number: SPEC P 028  Product no.: 4200301, 4200306, 4200321 |
|  | VIVAPHARM^®^ Alginate grade —PHU 175 | JRS Pharma  Batch Number: 4504005233  Manufacturing site: Landerneau, France |
| ^1^Buffer (Phosphate buffered saline (PBS) (10X), pH 7.4 without Calcium & Magnesium) | | Quality Biological  Cat#: 119-069-131  Lot#: 723633  Sterile filtered |
| ProvayBlue^®^ (methylene blue) Injection, USP  50 mg/10 mL (5 mg/mL) | | American Regent, Inc.  Lot number: E90011 |

¹ Components (mg/L): Na_2_HPO_4_ (anhydrous), KH_2_PO_4_, and NaCl

**Table S2:** The source of commercially available lifting agents used in this study.

| Normal Saline | BD PosiFlush™  Franklin Lakes, NJ 07417  SKU: 306547  GTIN: 00382903065462  Description: 10 mL pre-filled syringe |
| --- | --- |
| Starch (HEXTEND^®^) | Hospira Inc.  Lake forest IL-60045  LOT# 5965927  NDC: 0409-1555-64  Description: 500 mL Single-dose Flexible Container |
| ORISE™ Gel | Boston Scientific  300 Boston Scientific Way, Marlborough, MA 01752  Item# M00519201 |
| Eleview^®^ | Cosmo Pharmaceutical NV  Item#9000020  Description: Eleview^®^ 10 mL 5 ampoules |
| EverLift^®^ | LABORIE MEDICAL TECHNOLOGIES CORP.  Pease International Tradeport, 180 International Drive Portsmouth, NH 03801 USA.  Order# GIS-59  Description: EverLift^®^ Submucosal Lifting Agent 10 mL |
| BlueBoost™ | Microtech Endoscopy  2855 Boardwalk Drive  Ann Arbor, MI 48104  UPN/Order Number: SL62521  REF/Catalog Number: SML10  Description: BlueBoost™ Submucosal Injectable Solution, 10 mL  Package Units: 12/Box |
| Liftal^®^ K | Kaigen Pharma Inc.  2-5-14 Dosho-machi, Chuo-ku, Osaka 541-0045, Japan  Medical device name: 231ABBZX00031000  Description: Package: 5 bottles |

**Table S3:** Typical ingredients in a mucosal lifting gel.

| **Ingredient Class** | **Function/example** |
| --- | --- |
| Vehicle | Structure and medium for dispersion of ingredients  Example: Sterile water for injection |
| Viscosity modifier | Increase viscosity of gel  Example: poloxamer, JRS Na Alginate |
| Osmotic agent | Controls tonicity of formulation  Example: NaCl |
| Buffer | Controls the pH  Example: NaPO_4_^+2^ |

**Table S4**: The mathematical expressions, e.g., CIE XYZ, CIE L*a*b*, CIELCH and RGB color spaces to compare the vibrant blue color of gels.

|  |  | **ORISE^TM^ Gel** | **Eleview®** | **Everlift®** | **Blueboost^TM^** | **JRS Na Alg 0.6%** | **JRS Na Alg 0.8%** | **JRS Na Alg 1%** | **NovaMatrix MVG 1%** | **NovaMatrix MVM 1%** |
| --- | --- | --- | --- | --- | --- | --- | --- | --- | --- | --- |
| **CIE XYZ** | **X** | 33.9 | 37.0 | 27.7 | 38.8 | 21.7 | 19.3 | 21.0 | 45.1 | 49.9 |
|  | **Y** | 49.1 | 52.5 | 39.2 | 52.8 | 26.3 | 24.0 | 26.9 | 61.1 | 65.9 |
|  | **Z** | 78.5 | 90.3 | 91.7 | 98.2 | 66.3 | 62.5 | 61.0 | 100.0 | 101.8 |
|  | **f(X)** | 0.7 | 0.7 | 0.7 | 0.7 | 0.6 | 0.6 | 0.6 | 0.8 | 0.8 |
|  | **f(Y)** | 0.8 | 0.8 | 0.7 | 0.8 | 0.6 | 0.6 | 0.6 | 0.8 | 0.9 |
|  | **f(Z)** | 0.9 | 0.9 | 0.9 | 1.0 | 0.9 | 0.8 | 0.8 | 1.0 | 1.0 |
| **CIE Lab** | **L** | 75.5 | 77.6 | 68.9 | 77.8 | 58.4 | 56.1 | 58.9 | 82.4 | 84.9 |
|  | **a** | -39.6 | -38.0 | -34.0 | -32.9 | -14.6 | -16.6 | -20.3 | -33.9 | -31.2 |
|  | **b** | -22.4 | -27.4 | -43.4 | -32.5 | -42.1 | -42.7 | -36.5 | -25.6 | -22.4 |
| **CIE LCH** | **L** | 75.5 | 77.6 | 68.9 | 77.8 | 58.4 | 56.1 | 58.9 | 82.4 | 84.9 |
|  | **C** | 45.5 | 46.9 | 55.1 | 46.3 | 44.6 | 45.8 | 41.7 | 42.5 | 38.4 |
|  | **H** | 209.5 | 215.8 | 231.9 | 224.6 | 250.9 | 248.7 | 241.0 | 217.1 | 215.7 |
| **RGB Space** | **RV** | 0.0 | -0.1 | -0.2 | 0.0 | 0.0 | -0.1 | 0.0 | 0.0 | 0.1 |
|  | **GV** | 0.6 | 0.7 | 0.5 | 0.7 | 0.3 | 0.3 | 0.3 | 0.8 | 0.8 |
|  | **BV** | 0.7 | 0.9 | 0.9 | 1.0 | 0.7 | 0.6 | 0.6 | 1.0 | 1.0 |
|  | **R** | -156.2 | -192.8 | -530.6 | -145.3 | -104.1 | -182.8 | -121.2 | 43.3 | 88.5 |
|  | **G** | 207.2 | 212.7 | 188.3 | 211.6 | 151.4 | 146.3 | 154.8 | 224.6 | 230.3 |
|  | **B** | 224.5 | 239.5 | 244.0 | 249.6 | 212.1 | 206.7 | 203.7 | 250.2 | 251.5 |

**Figure S1:** Similarity heatmap produced from the ΔE matrix comparing color quantification across all formulations including our in-house prepared Na Alginate-based blue colored lifting gels in comparison to commercially available agents. Color Scale: Where Light Blue indicates low ΔE (closer to 0, better color accuracy), while Medium Blue indicates Moderate similarity values and Dark Blue signals high ΔE (greater color difference closer to the maximum, e.g., 38.2). This gradient helps visually distinguish how similar or different each pair of substances is.

### Stability study of lyophilized lifting gel

**Figure S2:** Lyophilization of injectable gel to obtain lyophilized cake to prolong stability of injectable gel and reconstitution in liquid prior to the procedure within a minute. Stability study of [A] NovaMatrix MVG 1% and [B] NovaMatrix MVM 1% lifting gel lyophilized in PBS and stored under accelerated condition (40 °C/75%RH) at t_0_ (initial), t_1_ (1 month) and t_6_ (6 months). Graphs showing the rheological properties of [C] and [D] NovaMatrix MVG -based lifting gel and [E] and [F] NovaMatrix MVM -based lifting gel reconstituted in water at t_0_ (initial), 1 month and 6 months of storage at 40 °C/75% RH as a function of shear rate vs shear at 25 °C and 37 °C respectively. For these graphs, the curvature indicated non-Newtonian behavior. Viscosity profile during upcurve and downcurve of [G] and [H] reconstituted NovaMatrix MVG 1% and [I] and [J] NovaMatrix MVM 1%-based lifting gel at t_0_ (initial), 1 month and 6 months of storage at 40 °C/75% RH [C] 25 °C and [D] 37 °C. Data points with error bars represent means ± standard deviations (n=3).

Conversely, on one hand, NovaMatrix MVG 1% gel lyophilized in PBS (0.8X, pH 6.5) and reconstituted in water stored under regular (25 °C / 60% RH) and intermediate (30 °C / 65% RH) conditions (see Figure S3) did not display any change in lyophilized cake appearance (intact, uniform and elegant light blue cake), reconstitution time (90 – 110 seconds), reconstituted solution color (clear, blue), viscosity, pH (6.5) and osmolality (265 – 275 mOsm/kg) between different timepoints, e.g., t_0_ (initial), t_1_ (1 month) and t_6_ (6 months). While on the other hand, NovaMatrix MVG 1% gel lyophilized in water and reconstituted in PBS (0.8X, pH 6.5) maintained under regular (25 °C / 60% RH) and intermediate (30 °C / 65% RH) (Figure S4) conditions exhibited no discernible changes in lyophilized cake appearance (intact, uniform and elegant sky-blue cake), reconstitution time, color, viscosity, pH, or osmolality throughout the study duration as shown in Table S5.

**Table S5:** Stability study of NovaMatrix MVG 1%-based lifting gel lyophilized in PBS (0.8X, pH 6.5) or lyophilized in water (both with methylene blue added) and reconstituted in water or PBS (0.8X, pH 6.5) respectively. Lyophilized cake visual appearance, reconstitution time, color (appearance), pH and osmolality as well as viscosity profile comparison at 25 °C and 37 °C at t_0_ (initial), t_1_ (1 month) and t_6_ (6 months) of stored under Regular (25 °C/60% RH) or Intermediate (30 °C/65% RH) conditions.

|  |  | t_0_ (initial) | | t_1_ = 1 month | | t_6_ = 6 months | |
| --- | --- | --- | --- | --- | --- | --- | --- |
| NovaMatrix MVG 1% in PBS lyophilized and reconstituted in Water | | | | | | | |
|  | Lyophilized cake appearance | Intact, uniform and elegant light blue cake | | Intact, uniform and elegant light blue cake | | Intact, uniform and elegant light blue cake | |
| Regular  25 °C/60% RH | Reconstitution time  [Seconds] | 98.7 ± 2.05 | | 103 ± 5.10 | | 96.7 ± 4.78 | |
|  | Color (gel appearance) | Clear, blue | | Clear, blue | | Clear, blue | |
|  | pH | 6.49 ± 0.005 | | 6.50 ± 0.005 | | 6.50 ± 0.012 | |
|  | Osmolality  [mOsm/kg] | 267.7 ± 1.89 | | 268 ± 0.82 | | - | |
|  |  | **25 °C** | **37 °C** | **25 °C** | **37 °C** | **25 °C** | **37 °C** |
|  | ¹Viscosity ± S.D.  [mPa∙s] | 62.5 ± 1.0 | 50.5 ± 1.6 | 57.3 ± 1.0 | 47.3 ± 1.2 | 58.8 ± 1.9 | 52.6 ± 2.1 |
|  | ²Viscosity ± S.D.  [mPa∙s] | 116.6 ± 4.5 | 79.2 ± 3.9 | 94.9 ± 0.8 | 73.8 ± 2.5 | 95.3 ± 2.4 | 84.2 ± 4.3 |
| Intermediate  30 °C/65% RH | Lyophilized cake appearance | Intact, uniform and elegant light blue cake | | Intact, uniform and elegant light blue cake | | Intact, uniform and elegant light blue cake | |
|  | Reconstitution time  [Seconds] | 98.7 ± 2.05 | | 105.3 ± 3.68 | | 97 ± 4.55 | |
|  | Color (appearance) | Clear, blue | | Clear, blue | | Clear, blue | |
|  | pH | 6.49 ± 0.005 | | 6.49 ± 0.009 | | 6.49 ± 0.005 | |
|  | Osmolality  [mOsm/kg] | 267.7 ± 1.89 | | 268.3 ± 1.25 | | - | |
|  |  | **25 °C** | **37 °C** | **25 °C** | **37 °C** | **25 °C** | **37 °C** |
|  | ¹Viscosity ± S.D.  [mPa∙s] | 62.5 ± 1.0 | 50.5 ± 1.6 | 55.6 ± 1.7 | 45.1 ± 1.5 | 62.0 ± 3.8 | 49.4 ± 1.1 |
|  | ²Viscosity ± S.D.  [mPa∙s] | 116.6 ± 4.5 | 79.2 ± 3.9 | 87.5 ± 2.6 | 68.5 ± 2.8 | 96.8 ± 8.0 | 73.1 ± 1.2 |
| NovaMatrix MVG 1% in Water lyophilized and reconstituted in PBS | | | | | | | |
| Regular  25 °C/60% RH | Lyophilized cake appearance | Intact, uniform and elegant sky-blue cake | | Intact, uniform and elegant sky-blue cake | | Intact, uniform and elegant sky-blue cake | |
|  | Reconstitution time  [Seconds] | 97.7 ± 1.25 | | 98 ± 0.82 | | 93.3 ± 4.03 | |
|  | Color (appearance) | Clear, blue | | Clear, blue | | Clear, blue | |
|  | pH | 6.50 ± 0.005 | | 6.50 ± 0.009 | | 6.50 ± 0.008 | |
|  | Osmolality  [mOsm/kg] | 269 ± 0.82 | | 268.7 ± 1.70 | | - | |
|  |  | **25 °C** | **37 °C** | **25 °C** | **37 °C** | **25 °C** | **37 °C** |
|  | ¹Viscosity ± S.D.  [mPa∙s] | 65.9 ± 1.0 | 45.5 ± 0.9 | 57.5 ± 0.4 | 43.2 ± 0.5 | 59.8 ± 0.4 | 54.7 ± 1.3 |
|  | ²Viscosity ± S.D.  [mPa∙s] | 122.5 ± 3.3 | 66.8 ± 3.1 | 88.5 ± 0.7 | 63.9 ± 2.4 | 91.8 ± 0.4 | 76.1 ± 1.3 |
| Intermediate  30 °C/65% RH | Lyophilized cake appearance | Intact, uniform and elegant sky-blue cake | | Intact, uniform and elegant sky-blue cake | | Intact, uniform and elegant sky-blue cake | |
|  | Reconstitution time  [Seconds] | 97.7 ± 1.25 | | 101 ± 3.56 | | 94.3 ± 5.25 | |
|  | Color (appearance) | Clear, blue | | Clear, blue | | Clear, blue | |
|  | pH | 6.50 ± 0.005 | | 6.49 ± 0.009 | | 6.49 ± 0.017 | |
|  | Osmolality  [mOsm/kg] | 269 ± 0.82 | | 268.3 ± 1.25 | | - | |
|  |  | **25 °C** | **37 °C** | **25 °C** | **37 °C** | **25 °C** | **37 °C** |
|  | ¹Viscosity ± S.D.  [mPa∙s] | 65.9 ± 1.0 | 45.5 ± 0.9 | 55.1 ± 1.3 | 44.8 ± 1.8 | 57.3 ± 1.0 | 49.6 ± 2.7 |
|  | ²Viscosity ± S.D.  [mPa∙s] | 122.5 ± 3.3 | 66.8 ± 3.1 | 83 ± 4.3 | 66.2 ± 0.6 | 85.6 ± 1.0 | 67.1 ± 1.0 |

¹Viscosity is measured at 1000 s^-1^ shear rate. ²Viscosity is measured at 142 s^-1^ shear rate.

**Figure S3:** Stability study of NovaMatrix MVG 1% lifting gel lyophilized in PBS and reconstituted in water stored under [A] 25 °C/60% RH and [B] 30 °C/65% RH at t_0_ (initial), t_1_ (1 month) and t_6_ (6 months). Graphs showing the rheological properties of [C] and [D] 25 °C/60% RH as well as [E] and [F] 30 °C/65% RH as a function of shear rate vs shear stress at 25 °C and 37 °C respectively. For these graphs, the curvature indicated non-Newtonian behavior. Viscosity profile during upcurve and downcurve of reconstituted NovaMatrix MVG 1%-based stored under [G] and [H] 25 °C/60% RH as well as [I] and [J] 30 °C/65% RH lifting gel at t_0_ (initial), 1 month and 6 months of storage at 25 °C and 37 °C respectively. Data points with error bars represent means ± standard deviations (n=3). All samples represent shear-thinning behavior.

Here it is important to highlight that NovaMatrix MVG 1% gels which were lyophilized in different medium, for example, in PBS (Figure S3) or water (Figure S4) lyophilized and stored in 60 mL vials under regular or intermediate conditions while during reconstitution of the above-mentioned lyophilized cakes in respective medium, 20 – 30 seconds more reconstitution time was required for gels than gels that were lyophilized and stored in 5 mL lyophilization vials (Figure S2). We conclude that 60 mL lyophilization vials have thicker walls which need slightly more time to get completely lyophilized or dried cake and then needs comparatively more reconstitution time because it needs more agitation by hand to completely reconstitute it as shown in Table S5. Nevertheless, gels that were lyophilized and stored in 60 mL vials necessitated an additional 20-30 seconds for reconstitution in comparison to those preserved in 5 mL vials, likely attributable to the thicker walls of the larger vials, which extended both drying and reconstitution duration.

**Figure S4:** Stability study of NovaMatrix MVG 1% lifting gel lyophilized in water and reconstituted in PBS stored under [A] 25 °C/60% RH and [B] 30 °C/65% RH at t_0_ (initial), t_1_ (1 month) and t_6_ (6 months). Graphs showing the rheological properties of [C] and [D] 25 °C/60% RH as well as [E] and [F] 30 °C/65% RH as a function of shear rate vs shear stress at 25 °C and 37 °C respectively. For these graphs, the curvature indicated non-Newtonian behavior. Viscosity profile during upcurve and downcurve of reconstituted NovaMatrix MVG 1%-based stored under [G] and [H] 25 °C/60% RH as well as [I] and [J] 30 °C/65% RH lifting gel at t_0_ (initial), 1 month and 6 months of storage at 25 °C and 37 °C respectively. Data points with error bars represent means ± standard deviations (n=3). All samples represent shear-thinning behavior.

The results derived from this investigation elucidate that lyophilized sodium alginate-based lifting gels display commendable stability when subjected to a spectrum of storage conditions, encompassing accelerated (40 °C / 75% RH), regular (25 °C / 60% RH) and intermediate (30 °C / 65% RH) conditions. Despite the adverse storage conditions, the gels sustained uniform reconstitution times, color, viscosity, and osmolality over a six-month timeframe. This finding is particularly salient considering that numerous pharmaceutical formulations are prone to alterations in physical properties when subjected to extreme thermal and humidity conditions. The minor discrepancies noted in viscosity between the 25 °C and 37 °C conditions align with the anticipated behavior of polymeric gels, which frequently exhibit temperature-dependent viscosity characteristics. However, the retention of stability by these gels across both temperature regimes is a promising indicator for their prospective application in clinical settings characterized by storage at room temperature and then application under physiological conditions.

The observation that lyophilized NovaMatrix MVG and NovaMatrix MVM gels preserved under accelerated conditions (40 °C / 75% RH) exhibited no substantial degradation in viscosity is especially significant. Also, we didn't see any difference in lyophilized cake appearance until 6 months. These findings imply that the formulation possesses sufficient robustness to endure more severe storage conditions, a crucial attribute for ensuring long-term product stability.

It is crucial to emphasize the observation that lyophilized gels contained within 60 mL vials demonstrated extended reconstitution durations in comparison to those encapsulated in smaller 5 mL vials. This phenomenon can be ascribed to the increased thickness of the vial walls, which may necessitate a prolonged duration for the thorough desiccation of the gel, thereby requiring additional agitation to attain complete reconstitution. This observation underscores the imperative to account for container dimensions in the formulation development and storage processes, particularly in instances where precise reconstitution times are of paramount importance.

In summary, the lyophilized sodium alginate-based lifting gels exhibited good stability across a spectrum of storage conditions (25 °C / 60% RH, 30 °C / 65% RH, and 40 °C / 75% RH) over a six-month period, with no notable alterations in critical physical attributes such as visual lyophilized cake appearance, reconstitution time, coloration, viscosity, pH, and osmolality. These results highlight the formulation's capability for long-term stability, even when subjected to accelerated conditions. Moreover, the minor fluctuations in reconstitution time attributed to vial size indicate that meticulous consideration of packaging design is vital for ensuring optimal performance.

### Ex vivo lifting hydrogel performance

EMR and ESD provide enhanced and more extensive resection margins compared to conventional polypectomy, with the capacity for en bloc excision of lesions. Both techniques utilize a submucosal injection of a lifting agent to facilitate the separation of tissue layers. Although saline and starch-based agents are routinely employed, their lifting duration (15–30 minutes) frequently proves inadequate for extended ESD procedures (approximately 45 minutes), thereby necessitating the need for repeated injections. To mitigate this limitation, an injectable gel formulated from Na Alginate designated as Generally Recognized as Safe (GRAS) by the Food and Drug Administration (FDA) was innovatively developed. In vitro investigations (including rheology, injectability, and stability assessments) pinpointed three optimal concentrations of JRS Na Alg 0.6%, 0.8% and 1% w/v while two optimal concentrations of NovaMatrix MVG and NovaMatrix MVM 0.8% and 1% w/v, which were subsequently scrutinized in an ex vivo model to evaluate lift quality and duration. Here, we evaluated the efficacy of the representative hydrogel formulations for their performance in key endoscopic tasks of submucosal injection and mucosal lifting. In this context, the porcine gastrointestinal tissue was used to determine the duration for which the gel formulations provide adequate lifting cushioning when injected for endoscopic resection (see Figure S5).

**Figure S5:** Schematic illustration of assessing the cushioning efficacy of lifting agent in the porcine gastrointestinal tissue.

**Figure S6:** Lifting persistency under simulation circumstances in the porcine model of Na Alginate based lifting gels in comparison to commercially available products at 0 min, 15 min, 30 min and 45 min; from left to right: Saline, Starch, ORISE™ Gel, Eleview^®^, EverLift^®^, BlueBoost™, Liftal^®^ K, JRS Na Alg 0.6%, JRS Na Alg 0.8%, JRS Na Alg 1%, NovaMatrix MVG 1% and NovaMatrix MVM 1%. Lifting gel depots are created, and cushion height and cushion breadth (circumference) increases after 15 min, 30 min and 45 min is measured.

**Lift persistency**

**Feel and snare**

**Injectability**

**Fundus**

**Body**

**Antrum**

**Lifting gel (1% MVG)**

**Stomach locations**

**Figure S7:** Graphical representation of injection of lyophilized-reconstituted lifting gel to the three different locations of stomach, namely, fundus, body and antrum. Then the injectability, lifting (including lifting persistency) and graspability with snare (hard or soft) and ability to snare evaluated and scored accordingly for all different formulations in the following table.

**Table S6:** The injectability, graspability with snare and lift of lifting gels in the ex vivo intact stomach model

| **Gel** | **Characteristic** | **Fundus** | **Body** | **Antrum** |
| --- | --- | --- | --- | --- |
| Saline | **^a^Injectability** | 3 | 3 | 3 |
|  | **^b^Graspability with snare** | 3 | 3 | 3 |
|  | **^c^Lifting persistency** | 1 | 1 | 1 |
| *Starch | **Injectability** | 3 | 3 | 3 |
|  | **Graspability with snare** | 3 | 3 | 3 |
|  | **Lifting persistency** | 1 | 1 | 1 |
| ORISE™ Gel | **Injectability** | 2 | 2 | 2 |
|  | **Graspability with snare** | 3 | 3 | 3 |
|  | **Lifting persistency** | 2 | 2 | 2 |
| Eleview^®^ | **Injectability** | 3 | 3 | 3 |
|  | **Graspability with snare** | 3 | 3 | 3 |
|  | **Lifting persistency** | 2 | 1 | 1 |
| Everlift^®^ | **Injectability** | 3 | 3 | 3 |
|  | **Graspability with snare** | 3 | 3 | 3 |
|  | **Lifting persistency** | 2 | 2 | 2 |
| BlueBoost^TM^ | **Injectability** | 3 | 3 | 3 |
|  | **Graspability with snare** | 3 | 3 | 3 |
|  | **Lifting persistency** | 2 | 1 | 2 |
| JRS Na Alg 0.6% | **Injectability** | 2 | 2 | 2 |
|  | **Graspability with snare** | 2 | 2 | 2 |
|  | **Lifting persistency** | 2 | 2 | 2 |
| JRS Na Alg 0.8% | **Injectability** | 2 | 2 | 2 |
|  | **Graspability with snare** | 2 | 2 | 2 |
|  | **Lifting persistency** | 3 | 3 | 3 |
| JRS Na Alg 1% | **Injectability** | 1 | 1 | 1 |
|  | **Graspability with snare** | 1 | 1 | 1 |
|  | **Lifting persistency** | 3 | 3 | 3 |
| NovaMatrix MVG 1% | **Injectability** | 2 | 2 | 2 |
|  | **Graspability with snare** | 2 | 2 | 2 |
|  | **Lifting persistency** | 3 | 3 | 3 |
| NovaMatrix MVM 1% | **Injectability** | 2 | 2 | 2 |
|  | **Graspability with snare** | 2 | 2 | 2 |
|  | **Lifting persistency** | 3 | 3 | 3 |

^a^Injectability: 3-Effortless, 2-Acceptable, 1-Strenuous. ^b^Graspability with snare: 3-Soft, 2-Firm, 1-Hard. ^c^Lifting persistency: 3-Optimal, 2-Adequate, 1-Suboptimal

^a^Injectability: 3-Effortless: minimal to no force required to push the agent into the syringe; 2-Acceptable: moderate force is required for the push; 1-Strenuous: difficult to push the agent into the syringe despite pushing with max. force.

^b^Graspability with snare: 3-Soft: Easy to grip the lift and snare; 2-Firm: Able to grip with snare after some slipping; 1-Hard: Unable to grip the lift due to slipping of snare.

^c^Lifting persistency: 3-Optimal: Able to appreciate the lift for >45 min; 2-Adequate: Able to appreciate the lift for about 30 minutes; 1-Suboptimal: Unable to appreciate the lift after 15 min of injection

**Table S7:** In vivo experiment evaluating the presence of submucosal cushion for at least 45 minutes when injected into live porcine gastrointestinal tissue.

| **Formulation** | **Cushion @ 15 min** | **Cushion @ 30 min** | **Cushion @ 45 min** |
| --- | --- | --- | --- |
| Saline (negative control) | Yes | No | No |
| Starch (positive control) | Yes | Yes | No |
| ORISE^TM^ Gel | Yes | Yes | Yes |
| Eleview^®^ | Yes | Yes | No |
| Everlift^®^ | Yes | Yes | No |
| Blueboost™ | Yes | Yes | No |
| JRS Na Alg 0.8% | Yes | Yes | Yes |
| JRS Na Alg 1% | Yes | Yes | Yes |
| NovaMatrix MVG 1% | Yes | Yes | Yes |

**Table S8:** Gross and histological changes at the injection site following autopsy and microscopic examination of the JRS Na Alginate-based lifting gel depot in pig stomach in day 3 and day 14 survival groups.

| **Specimen** | **Gross changes** | | | | **Histological changes** | |
| --- | --- | --- | --- | --- | --- | --- |
|  | **Discoloration** | | **Tissue integrity** | |  |  |
|  | **Day 3** | **Day 14** | **Day 3** | **Day 14** | **Day 3** | **Day 14** |
| Pig 1 | 2 | 1 | 1 | 1 | 2 | 2 |
| Pig 2 | 1 | 1 | 1 | 1 | 2 | 2 |
| Pig 3 | 1 | 1 | 1 | 1 | 2 | 2 |

**Discoloration: 1:** No change in the color; **2**: mild color changes along the edges; **3**: color change all along the edge; **4**: color change around the surrounding tissue. **Tissue integrity: 1:** No tissue disruption; **2**: Tissue erosion at the center of lift; **3**: Tissue erosion along the complete lift area; **4**: Tissue erosion beyond the lift area. **Histological scoring:** **1**: No inflammatory changes; **2**: Minor inflammatory changes; **3**: medium-Submucosal giant-cell type reaction; **4**: severe-Transmural granuloma formation.

**Table S9:** The injectability, feel and lift of lifting gels in the ex vivo the porcine gastrointestinal tissue

| **Score** | **Injectability** | **Feel** | **Lift** |
| --- | --- | --- | --- |
| 1 | Very difficult to completely inject the gel at the target site | Hard and difficult to snare | >50% decrease in height |
| 2 | Moderate difficulty requiring a longer time to deliver the gel | Hard but easy to snare | 40-50% decrease in height |
| 3 | Minimal difficulty during the injection but all the gel was delivered | Soft and easy to snare | 30-40% decrease in height |
| 4 | No difficulty with injection |  | 20-30% decrease in height |
| 5 |  |  | <20% decrease in height |

## **PCA modeling of data**

The loading plots as shown in Figure S8 with the first principal component (PC1) explained 91.79% of the overall variance, while the second principal component (PC2) accounted for 3.95% variance, while third principal component described for 2.83% of the total variance signifying that the majority of the variability within the dataset is encapsulated along the first principal component (see Figure S9). If examined in more detail, PC1 (91.79%) primarily explains the variance associated with viscosity, which shows a direct correlation with lift persistency and an inverse correlation with the percent decrease in height, increase in circumference of the cushion, and injectability. PC2 (3.95%) mainly accounts for the contribution of the flow behavior index (n), exhibiting an indirect relationship with the percent decrease in height and circumference increase, but a direct relationship with lift persistency. Finally, PC3 (2.83%) predominantly represents the variation attributed to the flow behavior (n) of the lifting gels, reflecting their shear-thinning characteristics.

Saline, Starch, ORISE™ Gel, Eleview^®^, EverLift^®^, and BlueBoost™ exhibited negative PC1 scores, reflecting less favorable characteristics such as lower viscosity and rapid decrease in lift height or increase in cushion circumference (Figure S9). In contrast, Liftal^®^ K and JRS Na Alg 0.6% displayed scores near zero, suggesting moderate lift performance. Formulations with PC1 scores between 5 and 10, including JRS Na Alg 0.8%, NovaMatrix MVG 1%, and NovaMatrix MVM 1%, demonstrated the most desirable lift persistency among all tested gels. These findings are consistent with endoscopist feedback obtained during in-house evaluations, where JRS Na Alg 0.8% and NovaMatrix MVG 1% were identified as the best-performing formulations in terms of viscosity and lift stability.

**A**

**B**

**C**

**Figure S8:** Loadings of the PCA model with in vitro variables, for example, the viscosity profiles of representative hydrogels, viscosity index (n), in conjunction with the other ex vivo study variables, namely, the percent decrease in lift height after a duration of 15-, 30 and 45-minutes, the increase in lift circumference after the same period, the persistence of lifting (Lift) and (D) the graspability of the lift with snare (feel) as well as injectability. (A) PC1 (91.79%), (B) PC2 (3.95) and PC3 (2.78%).

**Figure S9:** Principal Component Analysis (PCA) models utilizing the viscosity profiles of representative hydrogels, in conjunction with the variables of viscosity index (n), together with ease of injectability of formulation in tissue, percent lift height decrease, breadth increase as well as the graspability of the lift with snare. PC1 score showing distribution of lifting gel within 95% confidence interval.

We subsequently correlate the viscosity at highest shear rate of all formulations with the increase in lift height after a duration of 45 minutes (see Figure S10A), as well as the increase in lift circumference after the same period (see Figure S10B), or the persistence of lifting (see Figure S10C). Correlation analysis between viscosity and mucosal lift parameters demonstrated a strong relationship between rheological behavior and lift persistence. As shown in Figure S10, formulations with higher viscosity exhibited a slower decrease in lift height and circumference over 45 minutes, indicating enhanced mucosal lift stability. Conversely, low-viscosity formulations (e.g., saline and starch) rapidly lost lift height and circumference, reflecting poor lift maintenance. The close clustering of commercial products (BlueBoost^TM^, Eleview^®^, EverLift^®^ and ORISE^TM^ Gel) confirming their inferior lift characteristics than JRS Na Alginate-based formulations.

**A**

**B**

**C**

**Figure S10**: Correlation between viscosity and mucosal lift parameters for various formulations. The plots illustrate the relationship between viscosity (measured at a shear rate of 992.005 s⁻¹) and (A) height decrease after 45 min, (B) circumference increase after 45 min, and (C) lift (persistency). Higher viscosity formulations, such as JRS Na Alg 1%, JRS Na Alg 0.8% and NovaMatrix MVG 1%, demonstrated reduced height decrease and circumference expansion, indicating superior lift persistency. In contrast, low-viscosity formulations (e.g., Saline and Starch) exhibited rapid loss of lift. These results highlight the strong correlation between rheological properties and mucosal lift stability.
